# Full strength and toughness recovery after repeated cracking and healing in bone-like high temperature ceramics

**DOI:** 10.1038/s41598-020-75552-1

**Published:** 2020-11-04

**Authors:** Toshio Osada, Aiko Watabe, Joji Yamamoto, Johannes C. Brouwer, Cees Kwakernaak, Shingo Ozaki, Sybrand van der Zwaag, Willem G. Sloof

**Affiliations:** 1 Research Center for Structural Materials, National Institute for Materials Science, 1-2-1 Sengen, Tsukuba, Ibaraki 305-0047 Japan; 2 Faculty of Engineering, Yokohama National University, 79-5 Tokiwadai, Hodogaya, Yokohama 240-8501 Japan; 3 Department of Materials Science and Engineering, Delft University of Technology, Mekelweg 2, 2628 CD Delft, The Netherlands; 4 Faculty of Aerospace Engineering, Delft University of Technology, Kluyverweg 1, 2629 HS Delft, The Netherlands

**Keywords:** Materials science, Structural materials, Ceramics

## Abstract

Bones of humans and animals combine two unique features, namely: they are brittle yet have a very high fracture toughness linked to the tortuosity of the crack path and they have the ability to repeatedly heal local fissures such that full recovery of overall mechanical properties is obtained even if the local bone structure is irreversibly changed by the healing process. Here it is demonstrated that Ti_2_AlC MAX phase metallo-ceramics also having a bone-like hierarchical microstructure and also failing along zig-zag fracture surfaces similarly demonstrate repeated full strength and toughness recovery at room temperature, even though the (high temperature) healing reaction involves the local formation of dense and brittle alumina within the crack. Full recovery of the fracture toughness depends on the healed zone thickness and process zone size formed in the alumina reaction product. A 3-dimensional finite element method (FEM) analysis of the data obtained from a newly designed wedge splitting test allowed full extraction of the local fracture properties of the healed cracks.

## Introduction

Recent progress in the field of self-healing engineering materials^1,2^ has drastically altered our vision on component safety design in high tech and commodity products. When using current man-made structural materials, the damage evolution as a result of static, fatigue or impact loads causes a gradual or catastrophic but always irreversible reduction in the residual strength and this leads to a shortened product lifetime^3–5^. While the irreversibility of damage accumulation applies to all material families, it is particularly harmful when using brittle materials, since structural engineers can only compensate for this by using larger safety margins^3,4^ leading to accumulated weight increases for the entire structure.

Human bone, which is the main structural material in our body, is brittle and is continuously exposed to minor or mediocre overloads yet has to function even up to 100 years. Bone meets this requirement by a hierarchical and laminated substructure^6–10^, making the brittle material fracture in a ‘tough’ manner, together with ‘repeatable’ full recovery of strength and toughness by autonomous self-healing reactions involving reactive repair and remodelling stages^11,12^. The hierarchical laminated structure of the bone is responsible for the delocalisation of micro damage and results in fine crack patterns with a small separation distances between the crack faces. The healing reaction involves the formation of new solid bone filling up the cavity created by the fissures, with the required atoms being supplied to the damage site via the electrolyte surrounding the bone. Although the formation of new bone leads to a local discontinuity in material properties, nature succeeds in healing bone fracture in such a manner that the healed region has a comparable fracture resistance as the original bone structure and this local recovery of fracture resistance in a discontinuous material structure is the key aspect of the multi-healing capability of bone.

Early attempts to translate natural healing phenomena to man-made materials such as polymers^13,14^, concrete^15^, metals^16,17^ and high temperature ceramics^18–29^ focussed primarily on mechanisms to fill the crack and the resulting reduction in local fracture toughness and a reduced fracture strength was taken for granted. A notable exception was encountered in the fracture and high temperature healing of so-called MAX phase metallo-ceramics^30–35^, with compositions such as M_2_A_1_X_1_, M_3_A_1_X_2_ and M_4_A_1_X_3_, where M is an early transition metal, A is an A group element (mostly IIIA and IVA, like Al and Si) and X is either C or N. MAX phase material has an atomically layered structure of alternating mono-atomic layers of metallic atoms (A) and ceramic units (M_n+1_X_n_)^36,37^. The layered nano-structure mitigates the brittleness of the material and leads to enhanced *intrinsic* and *extrinsic* toughening^36,37^ including microcracking, crack bridging and crack deflection as also found in human compact bone^6–10^ and microscopically layered polymer-ceramics such as nacre^38,39^. As a result, MAX phase materials show a rising resistance to crack-growth with increasing crack length, the so-called R-curve behaviour^6,40–42^ and a zig-zag fracture path. As a result of their laminated structure the fracture behaviour of MAX phase is not unlike that of (fresh) bone.

While the MAX phase materials have a desirable fracture behaviour due to their natural layered microstructure, they have other advantages too. MAX phase ceramics are stable up to high temperatures and corrosion resistant^36,43,44^. The high thermal conductivity makes these ceramics thermal shock resistant^43^. Their static strength is maintained up to high temperatures, above which creep will become the limiting factor^37,43,45^. Also, MAX phase materials are easy machinable^43^. These properties make MAX phase materials attractive for high temperature applications, where the material is exposed to thermal cycles, mechanical loading and oxidation. These conditions are encountered in e.g. power or propulsion generation, raw material production, recycling facilities etc.

In a recent publication it has been shown that certain MAX phase ceramics, with compositions meeting six well defined physical criteria^32^, demonstrate autonomous healing when used in air and at a high temperature (typically > 1200 K)^30^. Like in bone, the healing reaction under these conditions is due to the filling of the crack cavity by a solid reaction product formed by a chemical reaction between atoms supplied by the MAX phase material and external external atoms (in this case oxygen), leading to the formation of a dense, well-adhering thermoelastically compatible filler layer with a composition different from that of the surrounding matrix^31^. The theoretical screening of all known MAX phase structures indicated that Ti_2_AlC should have the best healing ability and several experimental studies have indeed confirmed its excellent healability due to the formation of a dense and well-adhering Al_2_O_3_ layer in the former crack volume^31^. The reported occurrence of multiple complete or partial recovery of the room temperature strength upon sufficiently long exposure to hot, oxygen containing gasses^30,33–35^, indirectly indicates a complete or partial recovery of the local fracture mechanical properties in the healed crack region, notwithstanding the local heterogeneity in composition and mechanical properties.

In a previous study, the high-temperature crack filling behaviour in Ti_2_AlC was measured in-situ using synchrotron X-ray tomography^30^. These results clearly showed the filling of the microcracks by the local formation of alumina at the fracture surfaces all along the length of the crack. The alumina formed due to the diffusional supply of Al atoms from the MAX phase and the oxygen atoms supplied by the surrounding atmosphere (hot air). While mechanistically very relevant and informative, that study did not contain a detailed analysis of the mechanical properties including strength and toughness of healed parts. In the present work a combination of an experimental fracture mechanics set-up fitted with acoustic emission detectors and a companion inverse 3D FEM analysis is used to quantitatively determine the local (room temperature) fracture toughness and strength values of healed cracks for single and multiple cracking and healing events in the Ti_2_AlC MAX phase metallo-ceramics. A newly proposed elastoplastic-damage model including plasticity and cohesive-zone relationships is implemented to describe the elastoplastic-brittle fracture behaviour of both the original monolytic material as well as the facture behaviour of a heterogeneous sample containing a discrete former crack now filled with a different and intrinsically brittle material. The FEM model captures the experimental conditions of the fracture test used to induce initial cracking and re-cracking such that the reformed crack will preferentially follow the trajectory of the healed crack. The FEM model is suitable for inverse analysis of fracture strength and fracture toughness that are directly connected to the recovery phenomenon of metallo-ceramics. It will be demonstrated that the full and repeated recovery of the fracture toughness depends on the healed zone thickness and process zone size formed in the brittle alumina reaction product.

## Results

### Demonstration of repeated recovery of mechanical properties

**Figure 1:**
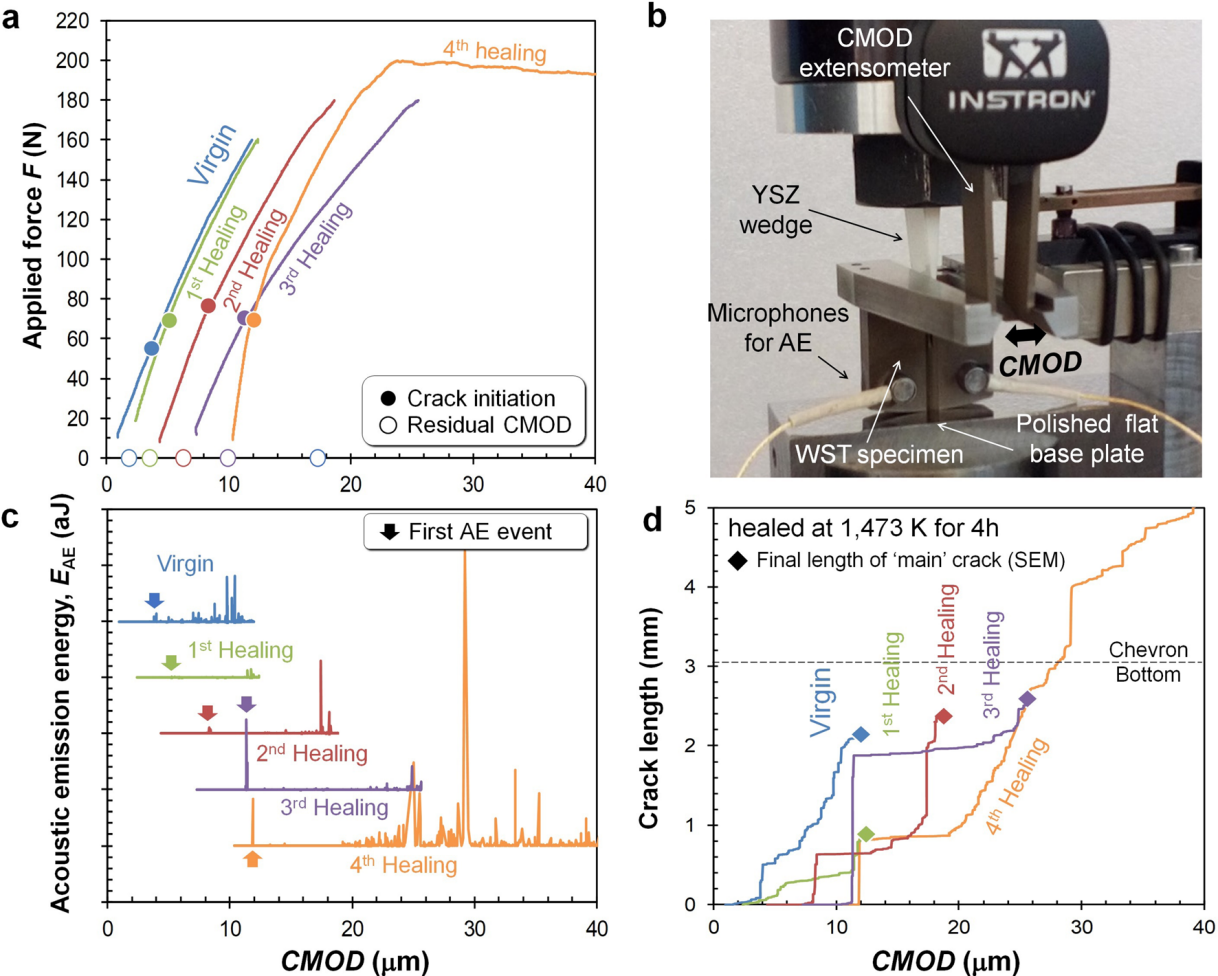
Recovery of mechanical properties by repeated cracking and healing in Ti_2_AlC: (**a**) Force versus displacement curves of multiple cracking and healing cycles. (**b**) Setup of the wedge-splitting test for controlled crack extension including acoustic emission monitoring, (**c**) Acoustic emission energy *E*_*AE*_ recorded during wedge splitting tests, (**d**) Crack length versus crack mouth opening displacement *CMOD* of multiple cracking tests.

Millimetre long cracks in a Ti_2_AlC sample with a hierarchical bone-like microstructure (see supplementary information Fig. S1) were repeatedly healed (Fig. 1 a-d) by exposure to air at a temperature of 1473 K and measuring the resulting fracture behaviour at room temperature. With the set-up and the data analysis protocol developed quantitative values for the key fracture mechanical properties of the sample (in its pristine and in a healed state) could be determined: its tensile strength (σ_F_) and its fracture toughness (*K*_IC_,). The cracks were initiated using a wedge-splitting test configuration (WST) and the force (*F*) and the crack mouth opening displacement (*CMOD*) were measured; see Fig. 1a,b (and supplementary information Fig. S2). To obtain the crack length as a function of the *CMOD* (Fig. 1d), the acoustic emission energy (*E*_AE_) was recorded during the enforced crack propagation; see Fig. 1c. The projected crack length was estimated from the cumulative acoustic energy *E*_AE_ and calibrated against the final crack length as measured with scanning electron microscopy (SEM); see supplementary information Fig. S3.

For the un-cracked virgin Ti_2_AlC sample, the *CMOD* increased linearly with the force applied to the wedge due to elastic distortion of the sample and the set-up. The first large acoustic emission event corresponding with the moment of crack initiation was detected at about 53 N. Next, a steady increase in the load on the wedge was required to cause stable crack growth within the chevron-notch. The slope of the force versus *CMOD* curve gradually decreased up to the peak force at about 160 N, since the specimen stiffness decreased due to propagation of the crack. This gradual, instead of abrupt, decrease is clear evidence of the relatively high toughness of the Ti_2_AlC MAX phase metallo-ceramic. Once the crack reached the bottom of the crack-guiding chevron (i.e. had reached a length of 3.05 mm) the load was removed by slowly withdrawing the wedge. The unloading step (not registered) only resulted in partial crack closure due to the zig-zag nature of the crack formed and the crack remained easily visible with the naked eye.

After creating a crack in the WST sample, it was removed from the set-up and healed for 4 h in a conventional lab furnace operating at 1473 K. This first healing treatment led to a thin layer of alumina filling the crack gap completely. Taking extremely good care of perfectly repositioning the sample in the set-up, the sample was re-cracked by driving the wedge back into the sample. The recorded force versus *CMOD* curve is similar to that for the virgin Ti_2_AlC; see Fig. 1a. No acoustic emission signal was detected until crack re-initiation occurred at about 72 N, which is higher than the load for first acoustical emission for the virgin material (53 N). Upon further loading the sample up to a load of 160 N a crack length of only 0.89 mm was reached as shown in Fig. 1d, which is rather short compared to that for the virgin material loaded to the same load, i.e. the healed sample has gained in fracture resistance. Next, the load was raised to 180 N and the crack was made to extend beyond the original crack length of the first loading cycle and reached a length of 2.37 mm. The recorded force versus *CMOD* curve is similar to that for the virgin and first-healed Ti_2_AlC case; see Fig. 1a. There is no clear indication at which load or *CMOD* value the crack tip left the healed crack path and entered the pristine material ahead of the healed crack.

This crack and healing of the WST sample was repeated (3^rd^ cycle). As for the first and second cycle, the force versus *CMOD* curves are similar; see Fig. 1a. The crack length after loading (2.59 mm) is slightly longer than that after the previous healing (2.37 mm); see Fig. 1d, and the crack initiation (at about 70 N) is about the same as after the previous healing; see Fig. 1a.

Finally, the WST sample was healed again but now loaded up to complete fracture. (4th loading cycle), The response of the healed sample, i.e. force versus *CMOD* curve and the point of crack initiation, is comparable with that after the previous healing cycles.

The results imply that the mechanical strength up to 3^rd^ healing cycles fully recovered due to full filling of the crack gap with a stronger material than the virgin Ti_2_AlC (having a fracture strength of about 275 MPa^36^), namely: alumina having a fracture strength of 800 MPa for a grain size of about 1.0 µm^46^. Furthermore, the repeated formation and healing of a dense alumina protective layer led to the higher force needed for crack initiation; see Fig. 4a,c,e. Finally, the rate of crack propagation at the beginning of the loading cycle increased slightly with increasing number of healing cycles. This is related to the continuous formation of the stiff and brittle alumina layer on top of the chevron tip; see Fig. 4a,c,e.

The slope of the force versus *CMOD* curves after crack initiation (Fig. 1a) for all healing cycles (1^st^ to 3^rd^), corresponding to stable crack growth behaviour (Fig. 1d), are the same as for the virgin Ti_2_AlC. This implies that the healed zone after the healing cycles exhibits the same level of apparent facture toughness as the virgin Ti_2_AlC, although the alumina filling the crack gap has a lower fracture toughness (~ 3.0 MPa·m^1//2^)^46^ than the Ti_2_AlC (~ 6.5 MPa·m^1/2^)^37^. Here, the apparent fracture toughness *K*_IC_ is given as follows:

1 $${K}_{\mathrm{IC}}={K}_{\mathrm{i}}+\Delta {K}_{\mathrm{R}}\left(\Delta a\right)$$

where *K*_IC_ is apparent fracture toughness of a material showing a R-curve behaviour^6,40–42^ with a crack length expansion Δ*a*. *K*_i_ and Δ*K*_R_(Δ*a*) are *intrinsic* and *extrinsic* fracture toughness, respectively. Higher toughness of Ti_2_AlC than alumina is caused by large Δ*K*_R_(Δ*a*) due to the elongated grains^37^. These findings suggest that the alumina-filled crack also exhibits an enhanced *extrinsic* toughness similar to that of Ti_2_AlC.

### Re-toughening behaviour during repeated healing

**Figure 2:**
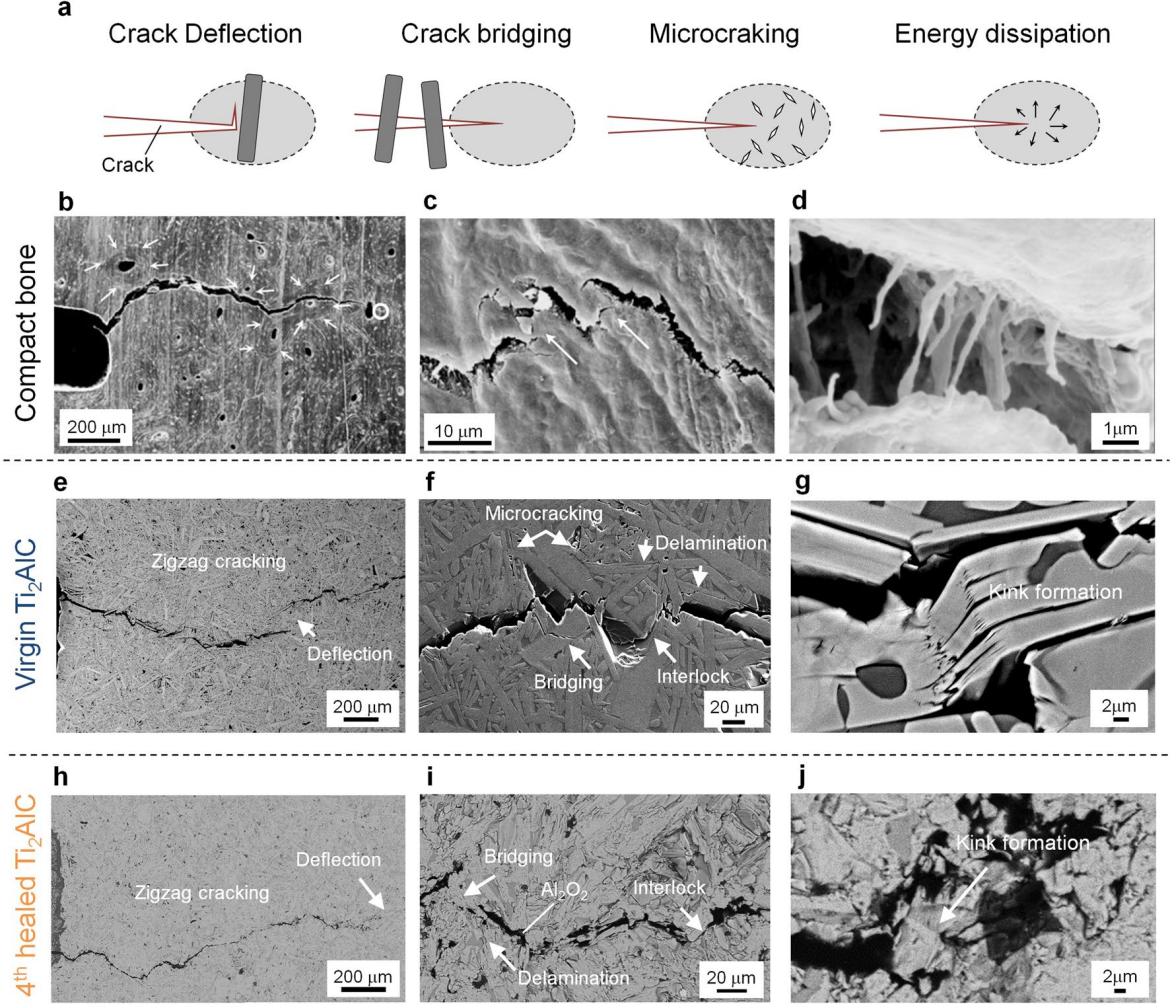
Crack propagation in Ti_2_AlC with bone-like hierarchical toughening structure. **(a**) Toughening mechanisms in compact bone. (**b**) Deflection by osteon, (**c**) Bridging and microcracking in bone, (**d**) Crack bridging by collagen fibrils. (**e**) Zig-zag cracking due to deflection by elongated grains, (**f**) A high-magnification image showing bridging, interlocking between fracture surfaces, (**g**) A high-magnification image showing kink band formation between fracture surfaces in the virgin Ti_2_AlC. (**h**) Zig-zag cracking, bridging, (**i**) interlocking between fracture surfaces. (**j**) Kink band formation during re-cracking in the 4^th^ healed Ti_2_AlC sample. Image (**b**–**d**) were taken from Nalla et al. ***Nature. Mater.*** 2 (2003) 164–168.

**Figure 3:**
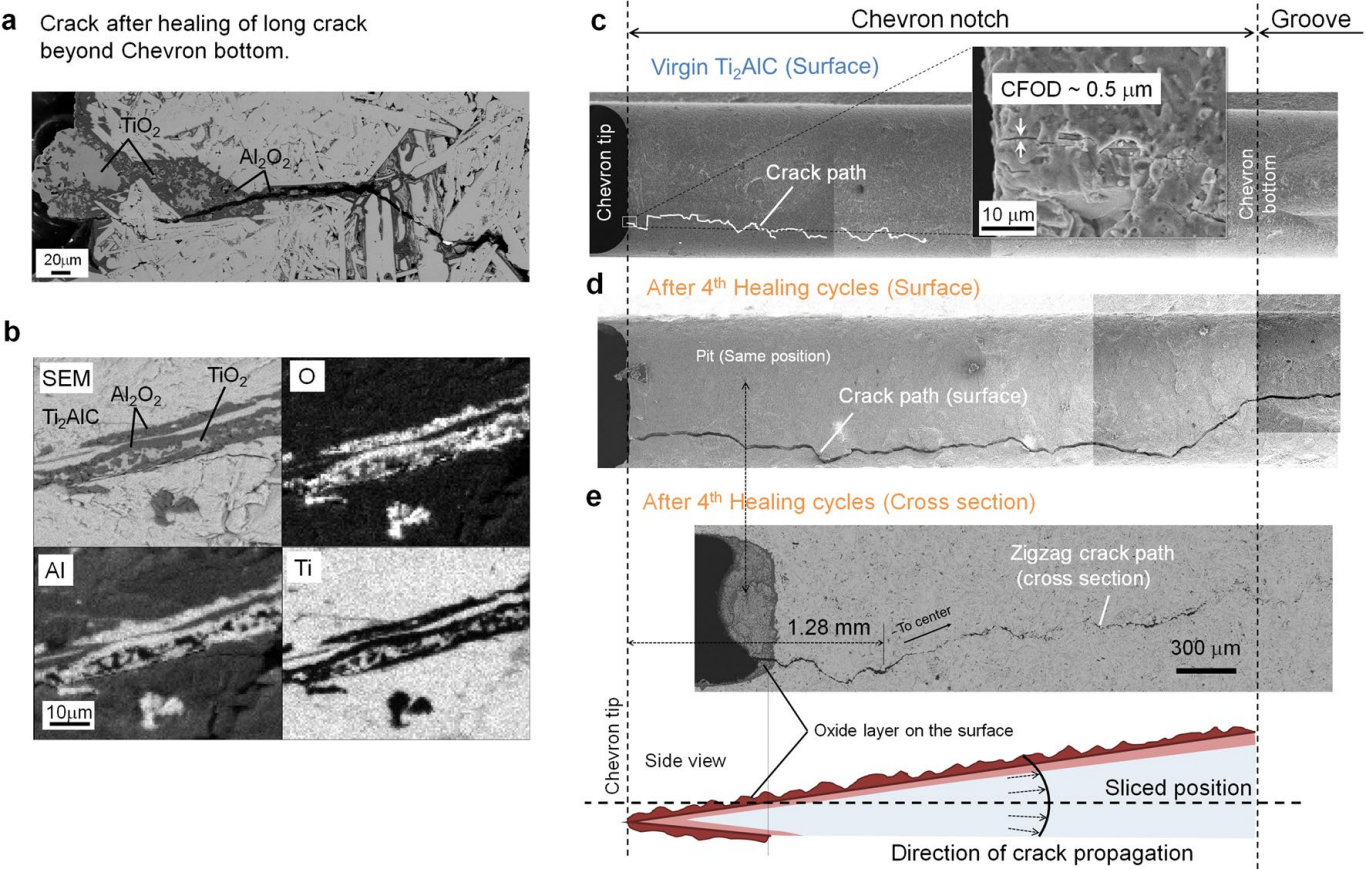
Filling of the crack gap and zig-zag crack path after multiple healing cycles of Ti_2_AlC. **(a**) SEM image of the crack initiation part at the chevron tip for healed specimen with crack length beyond the chevron bottom. (**b**) Element distribution maps showing Al_2_O_3_ and TiO_2_ formation in the crack healed part. **(c**) Crack propagation on the surface of the chevron notch for virgin Ti_2_AlC, and **d** after the 4^th^ healing cycle. **e** Zig-zag crack path in a cross section of the chevron notch in the specimen after 4^th^ healing cycle.

Bone-like zig-zag cracking rather than straight crack propagation (Fig. 2a–d)^7–10^ was seen in the Ti_2_AlC MAX-phase metallo-ceramics (Fig. 2e–g), which is a key observation given the intention to achieve full recovery of the fracture toughness after multiple healing cycles. Importantly, the same zig-zag cracking behaviour (Fig. 2 h–j) still occurs after multiple crack healing of the Ti_2_AlC MAX phase metallo-ceramics; see Fig. 3. All the corresponding toughening mechanisms, i.e., energy dissipation, microcracking, crack bridging and crack deflection (Fig. 2e–g) including interlocking, crack bridging and crack deflection as identified for human compact bone^7–10^ (Fig. 2b–d) are also observed in the chevron notch region of the virgin Ti_2_AlC. Plastic deformation of the MAX phase with atomic layered crystal structure is associated with energy dissipation due to dislocation motion and kinking; see Fig. 2g. Further, the elongated grains and laminated structure leads to extrinsic toughening mechanisms including microcracking, crack bridging and crack deflection (Fig. 2f), resulting in a rising crack-growth resistance (R-curve behaviour). Further, some kink formation near the crack gap was observed in the healed specimen; see Fig. 2j. It can be conceived that repeated energy dissipation by kink formation occurred in the healed specimen as well, because cyclic compressive reversible stress–strain loops were observed (see supplementary Fig. S5).

Alumina has fully filled the entire large crack gap (~ 5 µm) after oxidation-induced healing in air at 1473 K for 4 h even for a long crack extending beyond the bottom of the chevron (Fig. 3a,b), which is consistent with earlier observations^34^. Thus, crack gaps less than ~ 1 µm of the 1st to 4th healing are entirely filled by alumina similar to previous in-situ observations^30^. However, within the widest crack gap regions (~ 5 µm), also some TiO_2_ was formed in between the alumina deposit^34^; see Fig. 3a,b.

Also, at the surface of the WST sample a dense and closed alumina layer develops during the healing process. Upon loading of the sample, the crack is initiated in the alumina layer on the chevron tip. However, the crack initiation after each healing cycle occurred always from different sites of the chevron notch tip, which is evident from Fig. 3c,d. This implies that also the crack in the alumina layer on top of the chevron notch is healed; cf. Later section.

Once initiated, the crack propagates in a zig-zag mode in the healed zone of the Ti_2_AlC. Then, the crack follows the same path as the previous crack path. Fracture occurs mainly in the alumina rather than along the Al_2_O_3_/Ti_2_AlC interface; see Fig. 3a. Thus, after multiple healing cycles still a single crack path is observed in the cross section of the sample; see Fig. 3e.

The crack initiated at one side of the chevron notch (see Fig. 3c,d), eventually deflected towards the centre of the notch; see Fig. 3e. This is a feature of the design of the WST sample, which forces the crack to propagate in the stress concentrated part. Hence, the advantage of our WST method over other methods^18–27^ for evaluating crack healing behaviour, is that re-cracking in encouraged to occur in the healed zone of the material. Moreover, both the unique geometry of the chevron notch and the WST method in combination with a very stiff load frame ensures a slow crack growth rate within the sample which can be monitored easily using the acoustical sensors. Combined with reversed FEM calculations allows evaluation the R-curve behaviour and the toughening behaviour could be extracted, given the fact that the WST sample is not fully broking during the test, as is the case in the more common protocol of 3-point bend testing of indented samples^18–27^. By judiciously selecting the fracture conditions per test cycle, our method enables he extraction of quantitative fracture data including the R-curve behaviour of toughening, even for multiple ‘fracture and healing’ cycles.

### Novel aspects of the self-healing mechanism in alumina layer

**Figure 4:**
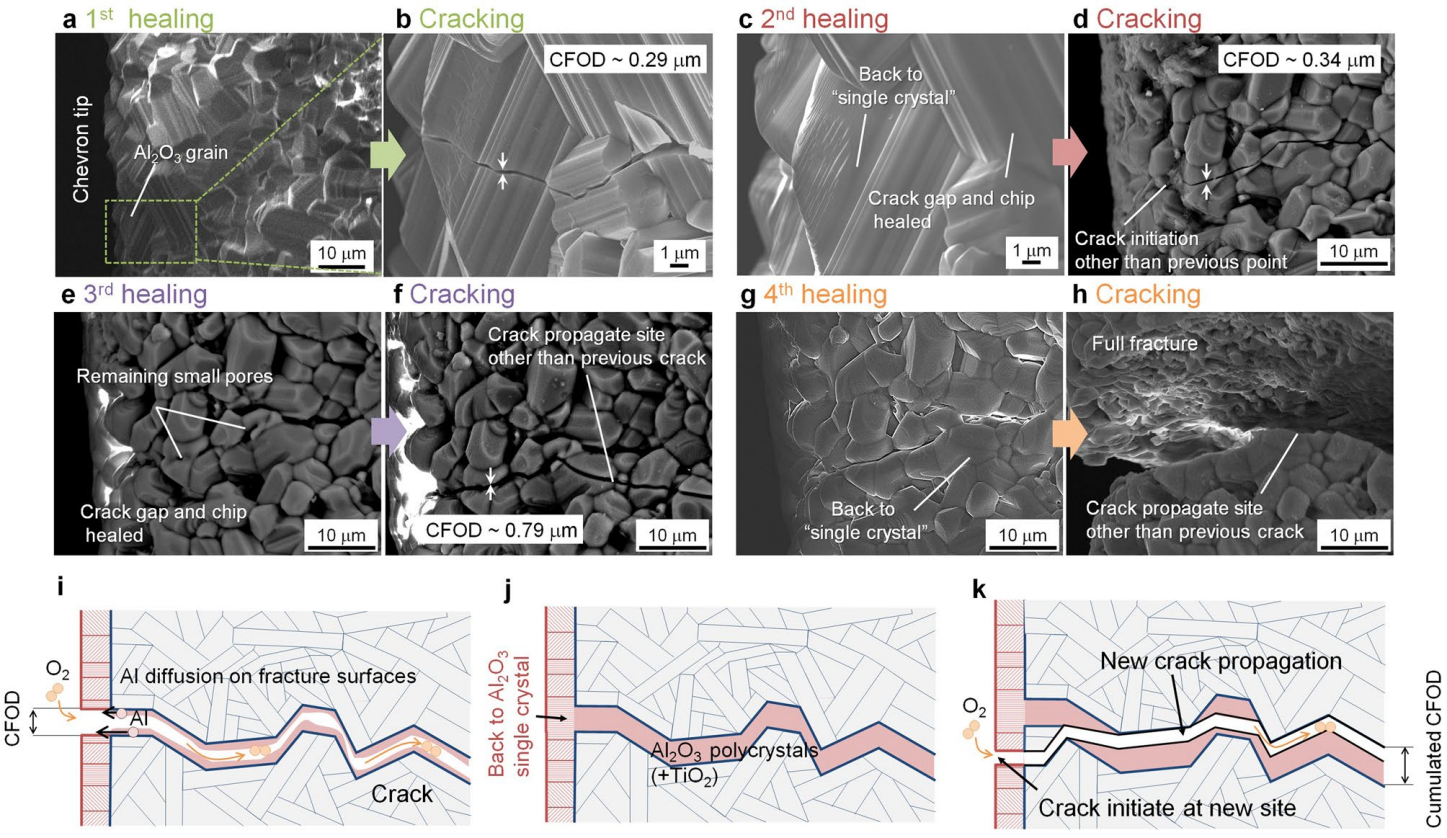
Repeatable healing of a crack in the Al_2_O_3_ protective layer. (**a**) SEM image showing a alumina formation after 1st healing cycle, and (**b**) Crack initiation at grain at the chevron tip. **c** Image after 2st healing cycle, and (**d)** cracking. (**e)** Images after 3rd healing cycle, and (**f)** cracking. (**g)** Images after 4th healing cycle, and (**h)** cracking. (**i,j)** and **(k)** Healing mechanism of oxide layer induced by Al atom diffusing outward and reacting with oxygen on fracture facets.

Surprisingly, crack healing also occurred in the protective Al_2_O_3_ layer formed on Ti_2_AlC (see Fig. 4a–h); which is paramount for full strength recovery and delayed crack initiation; cf. Previous section. After the first healing cycle, the crack initiated within coarse grained Al_2_O_3_ formed on top of the chevron tip and propagated mainly intergranular resulting in a gap with a crack-face opening distance CFOD of 0.3 µm; see Fig. 4a and b. At the initiation site, a trace of local plastic deformation can be seen; cf. Figure 4b. After the second healing cycle, the previous crack completely disappeared and the grain merged into the original single grain; see Fig. 4c. This crack healing in the alumina surface layer is also observed after the 3^rd^ healing cycle; see Fig. 4d–f.

The crack healing in the alumina is explained as follows. Fast outward diffusion of Al atoms on the fracture surfaces occurs from the Ti_2_AlC towards the alumina top layer driven by the chemical potential gradient of Al^47^; see Fig. 4i. Subsequently, the Al atoms react with oxygen from the environment and alumina grows epitaxially on both crack facets of α- Al_2_O_3_. Ultimately, the crack in the alumina closes and merges into a single grain; see Fig. 4j.

Once the oxide scale is formed after the first healing treatment, the thickness of the scale only increases slightly upon the next healing cycles. The growth of the coarse-grained alumina scale is mainly due to oxygen diffusion along the grain boundaries^33^. The surface ledges related to α- Al_2_O_3_ crystal structure changed a little and some rearrangement occurred of surface atomic structure near the plastic deformed zone; see Figs. 4b and c.

### Evaluation of local strength and toughness of healed zone

**Figure 5:**
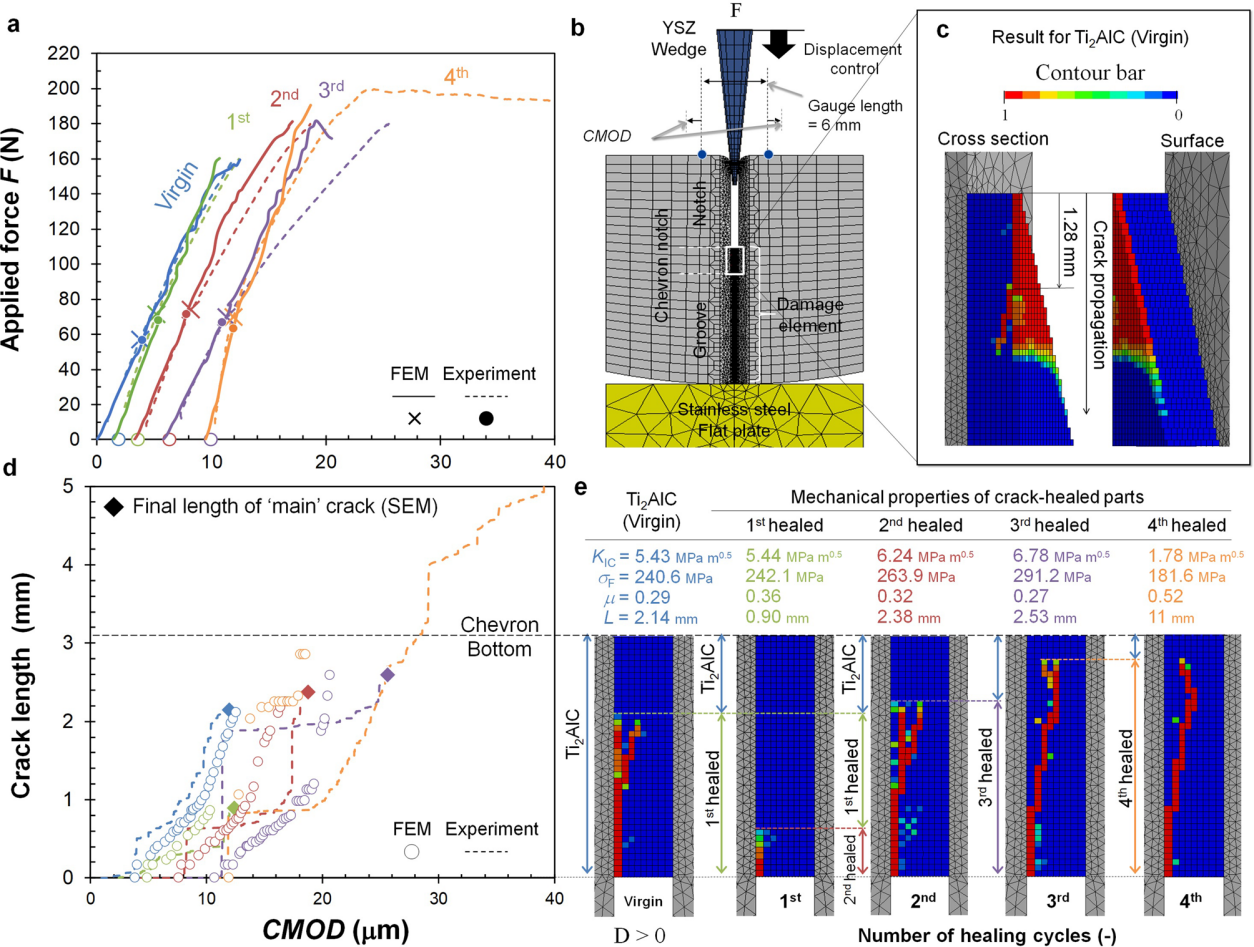
Mechanical properties of healed zone estimated by inverse 3D FE analysis. **(a)** Comparison between estimated and observed force versus crack mouth opening displacement (CMOD, **Dd**_**m**_) curves, (**b)** 3D finite element model. (**c) **Damage contours in surface and cross section after cracking of the virgin Ti_2_AlC chevron. (**d)** Comparison between estimated and observed crack length of multiple cracking tests. € Crack path, final length of main crack, strength and fracture toughness estimated by inversed 3D FEM analysis.

The fracture strength (σ_*F*_) and fracture toughness (*K*_IC_) recovery after crack healing are quantified by combining the fracture tests with the monitoring acoustic emission data (AE) and inversed fracture mechanical analysis using 3-dimensional finite element modelling (3D-FEM). To this end, a newly-developed elastic–plastic-damage (EPD) constitutive model was employed; see supplementary information Fig. S6. A comparison between the observed force and crack length versus *CMOD* curves, respectively, and the results of the 3D-FEM analyses is shown in Fig. 5.

The model calculations perfectly reproduced the measured force versus *CMOD* curves for the Ti_2_AlC virgin sample and after cracking and healing; see Fig. 5a. Moreover, the values obtained for the fracture strength and fracture toughness of the virgin Ti_2_AlC material are similar to reported values^37^, i.e. 222 MPa and 6.9 MPa m^1/2^, respectively. Further, the simulated crack initiation, growth and deflection behaviour during crack propagation (Fig. 5 c,d) are in good agreement with the experimental results; see Fig. 3c–e. The crack deflected at a similar depth from chevron tip and micro-cracks forming in addition to the main crack were also reproduced see Fig. 5e; and supplementary video S1.see Fig. 2f.

## Discussion

**Figure 6:**
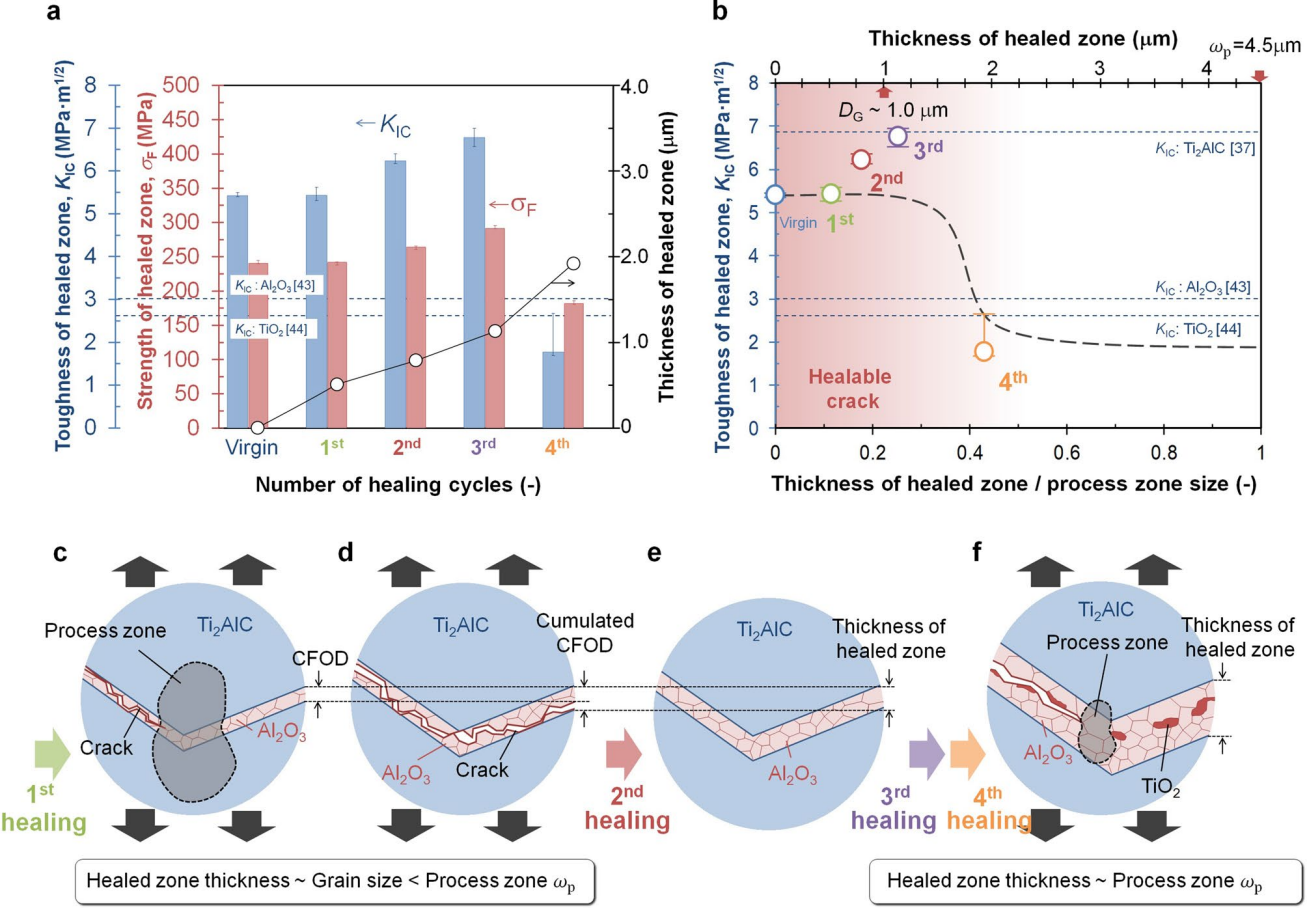
Full strength and toughness recovery after repeated crack healing: (a) Extracted local strength, toughness and thickness of healed zone of each healing cycles, (b) Critical ratio of thickness of healed zone /process zone size, and (**c-f)** schematic illustration of CFOD, thickness of healed zone, and the so-called process zone formation at healed zone filled by fine alumina grains.

Compared with the properties, as shown in Fig. 6 the fracture strength and fracture toughness after multiple healing (up to and including the 3rd healing) are similar or even higher than those of the pristine Ti_2_AlC (σ_F_ = 240 MPa and K_IC_ = 5.43 MPa m^1/2^). This is due to the formation of a well-adhering stiffer and stronger layer of alumina in the crack gap and on the surface. Thus, full strength recovery can be attained, even for a long crack with a large CFOD as long as the crack is filled with a stronger well-adhering filler material. The fracture toughness values of the healed Ti_2_AlC are higher than the values reported for α-Al_2_O_3_ (3.0 MPa m^1/2^)^46^ and TiO_2_ rutile (2.6 MPa m^1/2^)^48^ filler even when a relatively large crack in the chevron is healed. Thus, also full recovery of the toughness can be realized, due to a repeated toughening effect during re-cracking. After 4^th^ healing cycle, the strength decreased and the toughness of the healed zone reached the same magnitude as that for alumina or rutile. Then, the synergistic effect of the compound structure of an oxide filled crack in a MAX phase matrix is lost; see Fig. 6a.

It can be postulated that the degree of recovery of the toughness depends on the thickness of the healed zone. Here, the estimated thickness of healed zone is plotted in Fig. 6a, which is a summation of CFODs before each healing cycles; see Fig. 6c–f. The value of the CFOD after cracking of the virgin material and after the 1^st^, 2^nd^ and 3^rd^ cycles was ~ 0.5 µm (see Fig. 3c), 0.29 µm (see Fig. 4b), 0.34 µm (see Fig. 4d) and 0.79 µm (see Fig. 4f), respectively. Thus, the thickness of healed zone increased per healing and reached a value of 2 µm after the 4th healing cycle.

More importantly, it is also anticipated that the limit for full toughness recovery is related to the ratio the thickness of the healed zone to the process zone size ,ω_p_^49^ of the fine-grained alumina; see Fig. 6b–f. In a plain-stress state^49^ it holds that:

2 $${\omega }_{\mathrm{p}}=\frac{1}{\pi }{\left(\frac{{K}_{\mathrm{IC}}}{{\sigma }_{\mathrm{F}}}\right)}^{2}$$

which for a fine-grained Al_2_O_3_ filler can be estimated to be about 4.5 µm, considering a fracture strength σ_*F*_of 800 MPa and a fracture toughness *K*_IC_ of 3.0 MPa m^1/2^ for a 1.0 µm grain size^46^.

Values of the ratio of ω_p_ and thickness of healed zone is 0.4 for 4^th^ healing cycles. Then, the local fracture toughness of the healed zone is solely determined by that of the brittle alumina and rutile^48^; see Fig. 6b. The additional toughening mechanisms due to the deformation of Ti_2_AlC (cf. Figure 2) does not apply in this case. However, when the ratio is limited, the local fracture toughness of the healed zone is also determined by the surrounding of the zig-zag crack in the Ti_2_AlC involving toughening due to interlocking, crack bridging and crack deflection; see Figs. 2 a, h-j, because the thickness of healed zone up to and including the 3^rd^ healing cycle (see Fig. 4) are significantly smaller than the estimated process zone size ω_p_ of 4.5 µm. Hence, we conclude that it is important for full toughness recovery that the thickness of the healed zone is comparable to the grain size of the alumina filler. This leads to large plastic deformation pertaining to ω_p_ is 162 µm estimated from the FEM obtained values of σ_F_ is 240.6 MPa and K_IC_ is 5.43 MPa m^1/2^ of the virgin Ti_2_AlC surrounding of small gap filled by alumina.

It is interesting to note that full toughness recovery of tough metallo-ceramics could be achieved by filling with a ‘brittle oxide ceramic’. Recent advances in microstructure design approaches for brittle ceramics^6,36,37^ have led to significant improvements of their toughness. This will undoubtedly lead to a wider application of these materials for structural components. Among the various types of ceramics, MAX phases will be an attractive candidate in particular for high temperature structural components. Our findings demonstrate that the proposed toughening mechanisms, i.e., interlocking, crack bridging and crack deflection, also apply when brittle oxide ceramic filler material formed within the former crack still occurs in successive fracture and healing cycles. While in the current experiment healing and cracking were done at different temperatures (1473 K and 298 K) respectively, the microstructural observations suggest that complete recovery of the high temperature fracture mechanical properties is to expected too (provided of course that the net rate of crack propagation is lower than the rate for crack filling by oxidative reactions).

## Concluding remarks

The results presented here show that Ti_2_AlC MAX phase ceramics failing by a zig-zag fracture path and healing via the filling of the crack volume by a dense reaction product, behave in a similar manner as natural bone. In both cases not only a full recovery of the tensile strength, but more importantly of the fracture toughness is obtained. The companion FEM model captured this recovery of mechanical properties and gives clear guidelines on how to design these and other intrinsically self-healing high temperature ceramics. Future work should focus on studying cracking and healing both at the relevant high temperatures and on their kinetics such that accurate predictions can be made as to the life time of components made of or coated with Ti_2_AlC MAX phase.

## Methods

### Self-healing ceramics

High-purity of Ti_2_AlC composed of the elongated grain with high aspect ratio (average length of ~ 100 um, and thickness of ~ 10 um) having a bone-like structure (see supplementary information Fig. S1) was used in this study. Samples of Ti_2_AlC were synthesised by reactive sintering in a spark plasma sintering (SPS) furnace (HP D 25 SD, FCT Systeme GmbH, Germany). The Ti_2_AlC was sintered from Ti (100 μm, > 99.5%, TLS Technik GmbH &Co., Germany), Al (99.8%, 45 μm, TLS Technik GmbH &Co, Germany) and graphite (> 99.5, 6 μm, Graphit Kropfmühl AG, Germany) powder in a ratio of 0.85: 1.05: 1.15 at 1400 °C for 30 min. in a 40 mm diameter graphite mould under a uniaxial pressure of 50 MPa. These powders were mixed for 4 h using a Turbula T2C Mixer (Willy A. Bachofer, Switzerland) with 5 mm alumina balls. The phase purity of the samples was determined via X-ray diffraction using a Bruker D8 ADVANCE diffractometer (Bruker, Germany) in the Bragg–Brentano geometry with a graphite monochromator and Co Kα radiation. The recorded X-ray diffractrograms were processed with Bruker software DIFFRAC.EVA 4.1 software.

The Ti_2_AlC samples were machined by electro discharge machining (EDM) with a copper wire diameter of 0.1 mm into specially-designed wedge-splitting test specimen with a chevron-notch and a groove for controlled crack growth (see Fig. 5b). The tip of the chevron has a thickness of 0.75 mm, a height of 3.00 mm, and a tip angle of 45.2°, and is located at the bottom of the straight notch with a length of 10 mm length (detail drawing in supplementary information Fig. S2).

### Controlled crack extension test

The mechanical test setup for controlled crack growth is shown in Fig. 1b and in supplementary information Fig. S2. The fracture tests were performed with an electro-mechanical load frame (5500R series, Instron, USA). A crack was introduced by lowering a wedge made of rigid yttrium stabilized zirconia (YSZ) with a tip angle of 10° and a crosshead displacement rate of 0.01 mm/min. Then, the crack mouth opening displacement (*CMOD*) rate was less than 1.5 µm/min. To avoid any influence of snapback on the force–displacement curve, the *CMOD* was directly measured with a clip gauge extensometer placed on dedicated rigid legs mounted onto the specimen; see Fig. 1b. To realize a low yet constant friction between the specimen (before and after crack-healing treatment) and the wedge, their contact surfaces were fine polished to mirror finish with diamond suspension using 0.25 µm grains in the final step.

### Crack healing

The cracks generated in the Ti_2_AlC WST specimen were healed by annealing in air for 4 h at 1473 K in a horizontal alumina tube furnace with inner diameter of 80 mm (Carbolite TZF 17/600). Based on previous result on the oxidation of Ti_2_AlC^32–34^, this treatment ensures that the crack gap with having crack-face opening displacement (CFOD) less than 6 µm will be fully filled with mainly Al_2_O_3_. Note that the contact surfaces with the wedge of the crack-healed specimen were polished afterwards to mirror finish to avoid any influence of the surface alumina layer on the friction with the wedge.

### Microstructure observation

After each cracking and healing cycle, the crack length and microstructure were observed with optical microscopy (OM, Keyence, Japan, VHX-100), and scanning electron microscopy (SEM, JEOL Ltd., Japan, JSM-6500F) equipped with an energy dispersive spectrometer (EDS, Noran UltraDry) for X-ray microanalysis.

### Monitoring crack extension by acoustic emission

Acoustic emission was employed to monitor crack extension during the wedge splitting test^50^. Two microphones (type PICO S/N 4926 and 4928, nominal frequency 500 kHz) were mounted on the WST sample; see Fig. 1b. A Physical Acoustics Ltd. module (PCI-2, c channel 40 MHz 18 bit data-acquisition with ILS40 pre-amplifiers) was used to record the acoustic emission signal. The relation between the crack extension Δ*A* in chevron-notch and acoustic-emission energy *E*_AE_ has been established experimentally. The acoustic emission energy *E*_AE_ of a single wavelet is calculated from the amplitude *s*(*t*) of the recorded waveform having a duration *T*. This amplitude is normalized by the input impedance *Ω* of the measurement setup used, i. e.:

3 $${E}_{\mathrm{AE}}={\int }_{0}^{T}\frac{{\mathrm{s}}^{2}(t)}{\Omega }dt=\sum_{n=1}^{2048}\frac{{V}_{n}^{2}}{\Omega }\Delta t$$

In our setup, the time pulse length *T* consists of 2048 intervals of 0.5 μs duration each i.e. *T* = 1.024 ms. The impedance of the electronic setup used is 1 MOhm. In this calculation, a threshold energy level of 100 aJ was set to eliminate the noise on the acoustic emission signal. The time to crack initiation and the corresponding load were determined. The cumulative acoustic energy *E*_AE_ as a function *CMOD*; see Fig. 1c was determined through post-processing of the recorded acoustic events. From the total cumulative *E*_AE,_ and the final crack length observed with SEM, the crack length as function of *CMOD* was obtained as shown in Fig. 1d. The fractured surface area $\Delta A$, as determined after each cracking tests, is linear proportional with the corresponding cumulative Δ*E*_AE_; i.e.: Δ$\mathrm{A}=C{\Delta E}_{\mathrm{AE}}$, where *C* is a constant; see supplementary information Fig. S3.

### Inversed analysis of mechanical performances

To extract the important performances, i.e., strength and toughness after each damage and healing cycle, a FEM analysis approach was adopted with a newly-developed cohesive-force embedded unconventional elastoplastic-damage (EPD) constitutive model. In the EPD constitutive model, the elastic–plastic deformation process is described by the subloading surface model that belongs to the framework of unconventional plasticity^51^. The unconventional elastoplastic model assumes that the interior of the yield surface is not a purely elastic domain, and plastic strain also occurs by the change in the stress inside the yield surface. Hence, a smooth elastic–plastic transition can naturally be expressed during the loading process. To apply the EPD constitutive model to Ti_2_AlC, the von Mises yield criterion and nonlinear isotropic hardening law^51^ were adopted. Importantly, the stress–strain curve of Ti_2_AlC obtained from a cyclic compression test was used to determine parameters with respect to the elastoplastic deformation; see supplementary information Fig. S5a and Table S1.

Meanwhile, the damage process is described the relationship between cohesive-force and crack-opening, which is embedded in the subloading surface model. The cohesive-force embedded constitutive model provides equivalent performance to the traditional cohesive zoned model even in the relatively low numerical cost. In the newly developed EPD constitutive model, the fracture stress σ_*F*_ is prescribed by the combination normal stress σ and shear stress *τ* on the crack surface, i.e. ${\sigma }_{\mathrm{F}}=\sqrt{{\sigma }^{2}+\alpha {\tau }^{2}}$. After a damage initiation, the damage variable evolves by the equivalent strain κ. Note that, the softening induced mesh dependencies are handled by a characteristic length method^52^.

The relationship between stress rate and strain rate of EPD model is given as follows:4 where ${\varvec{\sigma}}$, ${\varvec{\varepsilon}}$, and ${{\varvec{C}}}^{ep}$ are the Cauchy stress tensor, the infinitesimal strain tensor, and the 4^th^ order-elastoplastic stiffness modulus based on the subloading surface model, respectively. The mechanical quantities in the fictitious undamaged configuration are denoted with: $\sim$. The damage variable *D* ($0\le D\le 1$) is given by:

5 $$D=1-\frac{{\kappa }_{0}}{\kappa }{exp}\left\{-\frac{{\sigma }_{\mathrm{F}}h(\kappa -{\kappa }_{0})}{{G}_{\mathrm{C}}}\right\}$$

where κ_0_ is the equivalent strain at damage initiation, *h* is the characteristic length of the finite element^53–55^. The critical energy release rate *G*_C_ is related to the toughness *K*. Based on linear fracture mechanics, it holds that *G*_C_ = *K*_IC_^2^/E; in which *K*_IC_ is the fracture toughness and *E* the Young’s modules. Here, *K*_IC_ is given by Eq. (1), hence *G*_C_ also includes intrinsic and extrinsic toughness^6,40–42^. Typical mechanical response of the EPD constitutive model is illustrated in Fig. S6b. The cohesive-force embedded damage model includes elastic, plastic, damage parts; see supplementary information Fig. S6b.

Commercial FEM software package LS-DYNA and its related user subroutine, *umatXX*^56^ was used in this study. A dynamic explicit method was taken to perform a numerical integration in the time domain. The actual setup and 3D geometry of the WST specimens as 1/2 model was used; see Fig. 5b. The part of chevron notch was discretized by eight-node hexahedral element with dimensions of 0.05 × 0.05 × 0.05 mm, while other parts were discretised by tetrahedral elements. The frictional contact condition was based on the penalty method and Coulomb’s law^56^ for contact boundaries between WST specimen, YSZ wedge and stainless-steel flat plate. In the calculations, the prescribed forced displacement of YSZ wedge into the vertical direction was applied as in the actual experiment. As values for the Young’s modulus 226 and 200 GPa were taken for WST specimen and YSZ wedge, respectively. For the Poisson ratio (*v*) and the yield stress of Ti_2_AlC, 0.23 and 160 MPa, respectively, were taken; see supplementary information Fig. S5a and S6b.

The friction coefficient µ, strength of material σ_*F*_, and critical energy release rate *G*_C_ of the Ti_2_AlC, were obtained from inverse analysis. First, the friction coefficient was determined from the initial slope of force-CMOD curve; see Fig. 1a. Then, the strength of material was retrieved by comparing the moment of damage initiation with the experimentally obtained crack-occurrence force as shown in Fig. 1a,c. After that, the critical energy release rate *G*_C_ was also resolved by comparing the analytically obtained crack length from number of damaged elements with the obtained experimental crack length as shown in Fig. 1 d. The optimum values for the friction coefficient, strength of the material and critical energy release rate were identified by the Levenberg–Marquardt method^57^. Error bars in Fig. 6a correspond to each increment of the objective values during minimization process.

The above procedure was sequentially applied to analyze the experimental data of the 1^st^ till the 4^th^ healed specimen, respectively. It is noted that, in the FE analysis of the healed specimen, only previously damaged parts corresponding to crack path were replaced with new material properties as target values of the inverse analysis. This allows to evaluate the strength and toughness recovery of healed part.

## Supplementary information

Supplementary Information 1.

Supplementary Video 1.

## References

[1] Hager MD (2010). Self-healing materials. Adv. Mater..

[2] van der Zwaag S, Brinkman E (2015). Self Healing Materials: Pioneering research in the Netherlands.

[3] Ashby MF (2005). Materials Selection in Mechanical Design.

[4] Sonsino CM (2007). Course of SN-curves especially in the high-cycle fatigue regime with regard to component design and safety. Int. J. Fatigue..

[5] Yoshida H (1996). Particle impact phenomena of silicon nitride ceramics. Phil. Mag. A.

[6] Wegst UGK (2014). Bioinspired structural materials. Nature Mater..

[7] Peterlik H, Roschger P, Klaushofer K, Fratzl P (2006). From brittle to ductile fracture of bone. Nature Mater..

[8] Thompson JB, Kindt JH, Drake B, Hansma HG, Morse DE, Hansma PK (2001). Bone indentation recovery time correlates with bone reforming time. Nature.

[9] Zioupos P, Currey JD (1994). The extent of microcracking and the morphology of microcracs in damaged bone. J. Mater. Sci..

[10] Nalla RK, Kinney JH, Ritchie RO (2003). Mechanistic fracture criteria for the failure of human cortical bone. Nature Mater..

[11] Taylor D, Hazenberg JG, Lee TC (2007). Living with crack: damage and repair in human bone. Nature Mater..

[12] Schindelera A, McDonalda MM, Bokkoa P, Little DG (2008). Bone remodeling during fracture repair: the cellular picture. Semin. Cell Dev. Biol..

[13] White SR (2001). Autonomic healing of polymer composites. Nature.

[14] Toohey KS (2007). Self-healing materials with microvascular networks. Nature Mater..

[15] Jonkers HM (2010). Application of bacteria as self-healing agent for the development of sustainable concreate. Ecol. Eng..

[16] Laha K, Kyono J, Sasaki T, Kishimoto S, Shinya N (2005). Improved creep strength and creep ductility of type 347 austenitic stainless steel through the self-healing effect of boron for creep cavitation. Metal. Mater. Trans..

[17] Fang H (2019). Self healing of creep damage in iron-based alloys by supersaturated tungsten. Acta Mater..

[18] Chu MC, Sato S, Kobayashi Y, Ando K (1995). Damage healing and strengthening behavior in intelligent mullite/ SiC ceramics. Fatigue Fract. Engng. Mater. Struct..

[19] Osada T (2017). A novel design approach for self-crack-healing structural ceramics with 3D networks of healing activator. Sci. Rep..

[20] Osada, T., Nakao, W., Takahashi, K., & Ando, K. Self-crack-healing behavior in ceramic matrix composites, *Adv. Ceram. Matrix Compos.* 410–441 (2014).

[21] Ando K (2002). Crack-healing behavior and high-temperature strength of mullite/ SiC composite ceramics. J. Eur. Ceram. Soc..

[22] Nakao W, Takahashi K, Ando K (2007). Threshold stress during crack-healing treatment of structural ceramics having the crack-healing the ability. Mater. Lett..

[23] Osada T, Nakao W, Takahashi K, Ando K (2009). Kinetics of self-crack-healing of alumina/silicon carbide composite including oxygen partial pressure effect. J. Am. Ceram. Soc..

[24] Ando K (2004). Crack-healing and mechanical behavior of Al_2_O_3_/SiC composites at elevated temperature. Fatigue Fract. Engng. Mater. Struct..

[25] Osada T (2007). Strength recovery behavior of machined Al_2_O_3_/SiC nano-composite ceramics by crack-healing. J. Eur. Ceram. Soc..

[26] Yoshioka S, Boatemaa L, van der Zwaag S, Nakao W, Sloof WG (2016). On the use of TiC at high-temperature healing particles in alumina based composites. J. Eur. Ceram. Soc..

[27] Yoshioka S, Nakao W (2014). Methodology of evaluate availability of self-healing agent for structureal ceramics. J. Intell. Mater. Syst. Stract..

[28] Derelioglu Z, Carabat AL, van der Song SM, Zwaag S, Sloof WG (2015). On the use of B-alloyed MoSi_2_ particles as crack healing agents in yttria stabilized zirconia thermal barrier coatings. J. Eur. Ceram. Soc..

[29] Maruoka D, Nanko M (2013). Recovery of mechanical strength by surface crack disappearance via thermal oxidation for nano-Ni/Al_2_O_3_ hybrid materials. Ceram. Int..

[30] Sloof WG (2016). Repeated crack healing in MAX-phase ceramics revealed by 4D in situ synchrotron X-ray tomographic microscopy. Sci. Rep..

[31] Kwakernaak C, Sloof WG (2018). Work of adhesion of interface between M_2_AlC (M = Ti, V, Cr) MAX phases and α-Al_2_O_3_. Ceram. Inter..

[32] Farle A, Kwakernaak C, van der Zwaag S, Sloof WG (2015). A conceptual study into the potential of M_*n*__+1_AX_*n*_-phase ceramics for self-healing of crack damage. J. Eur. Ceram. Soc..

[33] Song GM, Pei YT, Sloof WG, Ki SB, De Hosson JThM, van der Zwaag S (2008). Oxidation-induced crack healing in Ti_3_AlC_2_ ceramics. Scripta Mater..

[34] Yang HJ, Pei YT, Song GM, Hosson JThM (2013). Healing performance of Ti_2_AlC ceramics studied with in situ microcantiliver bending. J. Eur. Ceram. Soc..

[35] Song GM, Schnabel V, Kwakernaak C, van der Zwaag S, Sloof WG (2012). High temperature oxidation behaviour of Ti_2_AlC ceramics at 1200 °C. Mater. High Temp..

[36] Barsoum MW (2000). The M_(__*N*__+1)_AX_(__*N*__)_ phases: a new class of solids; thermodynamically stable nanolaminates. Prog. Solid. State. Chem..

[37] Barsoum MW, Radovic M (2011). Elastic and mechanical properties of the MAX phases. Annu. Rev. Mater. Res..

[38] Okumura K, De Gennes PG (2001). Why in nacre strong? Elastic theory and fracture mechanics for biocompisites with stratified structure. Eur. Phys. J. E.

[39] Song F, Soh AK, Bai YL (2003). Structural and mechanical properties of the organic matrix layers of nacre. Biomaterials.

[40] Lawn B (1993). Fracture of Brittle Solids.

[41] Awaji H (2000). Toughening mechanisms of structural ceramics. J. Ceram. Soc. Japan.

[42] Steinbrech RW (1992). Toughening mechanisms for ceramics materials. J. Eur. Ceram. Soc..

[43] Barsoum, M.W., Properties of machinable ternary carbides and nitrides, Wiley-VCH Verlag GmbH, Weinheim.

[44] Barsoum, M.W., Physical properties of the MAX phases, in: K.H.J.B. Editors-in-Chief: Robert, W.C., Merton, C.F., Bernard, I., Edward, J.K., Subhash, M., Patrick, V. (eds.) Encyclopedia of Materials: Science and Technology (Second Edition), 1–11 Elsevier, Oxford, (2006).

[45] Sun ZM (2011). Progress in research and development on MAX phases: A family of layered ternary compounds. Inter. Mater. Rev..

[46] Miyahara N (1994). Effect of grain size on strength and fracture toughness in alumina. JSME Int. J. A.

[47] Smialek JL (2015). Oxygen diffusivity in alumina scales grown on Al-MAX phases. Corr. Sci..

[48] Kim H-C (2006). Fabrication of ultra-fine TiO_2_ ceramics by high-frequency induction heated sintering method. J. Ceram. Process. Res..

[49] KinLoch AJ, Shaw SJ (1981). The fracture resistance of a toughened epoxy adhesive. J. Adhes..

[50] Farle (2018). Determination of fracture strength and fracture energy of (metallo-) ceramics by a wedge loading methodology and corresponding cohesive zone-based finite element analysis. Enging. Fract. Mech..

[51] Hashiguchi K (2017). Foundations of elastoplasticity: Subloading surface model.

[52] Oliver J (1989). A consistent characteristic length or smeared cracking models. Int. J. Numer. Meth. Eng..

[53] Ozaki S, Osada T, Nakao W (2016). Finite element analysis of the damage and healing behavior in self-healing ceramic materials. Int. J. Solids Struct..

[54] Ozaki S, Aoki Y, Osada T, Takeo K, Nakao W (2018). Finite element analysis of fracture statistics of ceramics: effects of grain size and pore size distributions. J. Am. Ceram. Soc..

[55] Takeo K, Aoki Y, Osada T, Nakao W, Ozaki S (2019). Finite element analysis of the size effect on ceramic strength. Materials.

[56] LSCT, LS-DYNA User’s Manual. *LSCT Singapore*, (2019).

[57] Yamashita N., Fukushima M., On the rate of convergence of the Levenberg-Marquardt method. In: Alefeld G., Chen X. (eds) Topics in Numerical Analysis. Computing Supplementa, 15. Springer, Vienna, (2001).

