# Supplementary material for: Full strength and toughness recovery after repeated cracking and healing in bone-like high temperature ceramics
Source: Sci Rep. 2020 Nov 4;10:18990. doi: 10.1038/s41598-020-75552-1 (PMC7643164; doi:10.1038/s41598-020-75552-1)
Supplement: Supplementary file 1 — Supplementary Information 1. [file 41598_2020_75552_MOESM1_ESM.pdf]

SUPPLEMENTARY INFORMATION for

Full Strength and Toughness Recovery after Repeated Cracking and Healing in Bone-like High-Temperature Ceramics

Toshio Osada[1*], Aiko Watabe[2], Joji Yamamoto[2], Johannes C. Brouwer[3], Cees Kwakernaak[3], Shingo Ozaki[2], Sybrand van der Zwaag[4] and Willem G. Sloof [3]

[1]Research Center for Structural Materials, National Institute for Materials Science, 1-2-1 Sengen, Tsukuba, Ibaraki 305-0047, Japan.

[2]Faculty of Engineering, Yokohama National University, 79-5 Tokiwadai, Hodogaya, Yokohama 240-8501, Japan.

[3]Department of Materials Science and Engineering, Delft University of Technology, Mekelweg 2, 2628 CD Delft, The Netherlands.

[4]Faculty of Aerospace Engineering, Delft University of Technology, Kluyverweg1, 2629 HS Delft, The Netherlands.

*Correspondence addressed to T.O. (email: OSADA.Toshio@nims.go.jp).

Bone-like hierarchical structures and toughening mechanisms

Ti_2AlC does not only have an excellent self-healing functionality but also has a hierarchical structure similar to that of human compact bone (Figs S1 a-e). The presence of large elongated grains with a high aspect ratio (Fig. S1f) leads to crack bridging and crack deflection causing toughening on a microscopic scale and the same mechanisms apply to osteon and its twisted arrangement (Fig. S1a and b). Similarly, the planar delamination within the Ti_2AlC grains (Fig. S1g) can lead to microcracking, just like the fibres formation upon longitudinal splitting of the osteon (Fig. S1c). Importantly, the Ti_2AlC MAX-phase has a layered crystal structure (Fig S1i) consisting of mono-atomic thick Al layers interspersed by layers of two titanium carbide like unit cells, enabling energy dissipation by dislocation motion and kinking (Fig S1h). This results in an additional toughening

mechanism similar to that found in collagen fibrils composed of stacked collagen molecules (Figs S1d and e).

Eventually, in the fracture surface of broken Ti_2AlC samples, traces of all toughening mechanisms were observed, which are similar to the mechanisms identified in fracture of human compact bone, i.e., crack deflection, crack bridging, microcracking, and localised energy dissipation (Figs 2 a-d).

Wedge splitting test

Controlled crack extension test is realized with a Wedge Splitting Test (WST) shown in Fig. S2a. The geometry of the WST specimen is illustrated in Fig. S2b. The chevron notch tip is located at a depth of 10 mm from the top of the specimen. To achieve a slow and straight crack propagation mode, a chevron with a tip angle of 22.6° and a length of 3 mm from top to bottom, and 90° guide-grooves on both side of the sample were electromachined. The actual length of the chevron was 3.04 mm. The bottom of the sample is semi-circular with a radius of curvature of 52 mm to allow specimen self-alignment upon insertion of the wedge. The sample rests on a planar and smooth hardened surface.

Acoustic emission

Acoustic emission[S5-S6] is employed to monitor crack extension during the wedge splitting test. Two miniature microphones (type PICO S/N 4926 and 4928, nominal frequency 500 kHz) are mounted on the WST sample; see Fig. 1b and Fig. S2a. The acoustic emission energy release rate is proportional to the release rate of the micro-mechanical energy stored at the crack front. [S6];

$$\frac{\partial E_{\mathrm{MM}}}{\partial \mathrm{t}} = \alpha \frac{\partial E_{\mathrm{AE}}}{\partial \mathrm{t}} \qquad \text{(S1)}$$

Where α is a proportionality constant and t denotes time, respectively.

The acoustic emission energy E_{AE} of a single wavelet is calculated from the amplitude $s(t)$ of the recorded waveform having a duration T. This amplitude is normalized by the input impedance Ω of the measurement setup used, i. e.:

$$E_{\mathrm{AE}} = \int_0^T \frac{s^2(t)}{\Omega} dt = \sum_{n=1}^{2048} \frac{V_n^2}{\Omega} \Delta t \qquad \text{(S2)}$$

In our setup, the time pulse length T consists of 2048 intervals of 0.5 μs duration each i.e. T=1.024 ms. The impedance of the electronic setup used is 1 MOhm. The range of energies recorded varies between 1 aJ and 10^9 aJ.

The fracture energy E_{MM} is derived using the stress intensity concept of linear elastic fracture mechanics (LEFM). Following Irwin's modification of the original Griffith's energy relation[S7], the strain energy release rate G_{C} is written as:

$$G_{\mathrm{C}} = -\left[\frac{\partial E_{\mathrm{MM}}}{\partial \mathrm{A}}\right]_{z=\mathrm{constant}} \quad \text{(S3)}$$

The above equation relates the energy E_{MM} required to increase the free surface of a solid by an area A, to the strain energy release rate G_C at a given constant displacement z. Hence, the relationship between E_{MM}, A and E_{AE} can be written as:

$$\frac{\partial E_{\mathrm{MM}}}{\partial \mathrm{t}} = \frac{\partial A}{\partial \mathrm{t}} G_{\mathrm{C}} = \alpha \frac{\partial E_{\mathrm{AE}}}{\partial \mathrm{t}} \quad \text{(S4)}$$

Thus, the extension of the fracture surface area ΔA is proportional to the cumulative acoustic signal ΔE_{AE} over the period of crack growth:

$$\Delta A = \frac{\alpha}{G_{\mathrm{C}}} \Delta E_{\mathrm{AE}} = C\, \Delta E_{\mathrm{AE}} \quad \text{(S5)},$$

where C (= α/G_C) is a constant corresponding to the slope of a plot of ΔE_{AE} versus ΔA

Fig. S3 shows the relation between ΔE_{AE} and ΔA for cracks in the pristine material and after healing. The area expansion ΔA is estimated from the final length of the crack observed with scanning electron microscopy and assuming a straight and perpendicular crack front. The data of both the virgin Ti_2AlC specimens and the crack-healed specimens both can be described with Eq. (S4), and have the same slope C =3.318×10^{-7} mm^2/aJ, as shown in Fig. S3.

Cyclic compression test

Uniaxial cyclic compression tests were carried out on Ti_2AlC samples to evaluate the loading-unloading stress-strain curves while monitoring the AE activity; see Fig. S4a. The cylindrical specimen with a height of 10 mm and diameter of 6 mm were prepared by electro discharge machining (EDM); see Fig. S4b. The strain of the specimen was measured directly with an extensometer. The strain rate was set to 1×10^{-4} /s.. Cylindrical compression heads of high-hardness ball bearing steel (102Cr6) were placed on both side of the specimen. The planar and parallel contact surfaces were well polished to mirror finish with diamond suspensions (up to 0.25 μm diamond grains). Multiple loading-unloading cycles were imposed with strain reversal at strains of , 0.2 %, 0.2 % (again), 0.4 %, 0.6% and to fracture, respectively; see Fig. S5a. The deformed microstructure after sample failure and cross-sectioning was observed with SEM; see Fig. 5b-e.

Plastic deformation in Ti_2AlC

Cyclic compressive stress –strain curves clearly show traces of plastic deformation above a strain of ~ 0.05 % and a stress of ~100 MPa; see Fig. S5a. The compression strength was measured to be 600 MPa at a strain of about 0.65 %. Above this strain, the stress decreased but a large AE activity was recorded. The applied load dropped significantly at a strain of 1.0 % because of specimen

(shear) failure. From the initial slope of each unloading curve, the Young's modulus of Ti_2AlC was determined to be 276 ± 11 GPa. In the 1st to the 4th compression cycle, the stress vs strain loops show the typical shapes for MAX phase materials [S8-S9] deforming via kinking and delamination. In the microstructure of the fractured specimen (see Fig. S5b-e), traces of plastic deformation and residual damages can be observed. In the low shear strain region marked 'c' in Fig. S5b, Incipient Kink Bands (IKB) can be observed within grains oriented close to 45° with respect to loading direction. In the medium and high shear strain regions, 'd' and 'e'in Fig S5b, grain boundary (GB) cracking and crack coalescence can be observed. The data obtained from the cyclic stress-strain curves with loops and residual strain serve as input data for the FE analysis.

Constitutive model in FEM

As mentioned in the main text, an unconventional cohesive-force embedded elastoplastic-damage constitutive model is adopted within the framework of the continuum damage theory[S10]. The elastoplastic constitutive relation is formulated rigorously in terms of the effective stress in the fictitious undamaged configuration. The constitutive equations are formulated consistently in terms of the effective stress and which is then transformed into the actual damaged configuration. Therefore, the elastoplastic-damage constitutive model is newly developed by incorporating the subloading surface concept[S11, S12, S13] as well as the cohesive zone relation embedded isotropic damage formulation[S14]. Note that, henceforth, the mechanical quantities in the fictitious undamaged configuration are denoted with:∼.

The relationship between the current stress tensor $\boldsymbol{\sigma}$ and the effective stress tensor $\utilde{\boldsymbol{\sigma}}$ is given by

$$\boldsymbol{\sigma} = (1 - D)\utilde{\boldsymbol{\sigma}} \tag{S6}$$

where the damage variable D $(0 \leq D \leq 1)$ describes the magnitude of damage (D=0 corresponds to the non-damaged state, while D=1 represents a perfectly fractured state). The stress rate $\dot{\boldsymbol{\sigma}}$ is given by

$$\dot{\boldsymbol{\sigma}} = (1 - D)\dot{\utilde{\boldsymbol{\sigma}}} - \dot{D}\utilde{\boldsymbol{\sigma}} = (1 - D)\dot{\utilde{\boldsymbol{\sigma}}} - \dot{D}\frac{\sigma}{(1-D)} \tag{S7}$$

The $\dot{\utilde{\boldsymbol{\sigma}}}$ is evaluated by the elastoplastic constitutive equation.

The infinitesimal strain rate tensor $\dot{\boldsymbol{\varepsilon}}$ is additively decomposed into the elastic part $\dot{\boldsymbol{\varepsilon}}^e$ and the plastic part $\dot{\boldsymbol{\varepsilon}}^p$, as follows:

$$\dot{\boldsymbol{\varepsilon}} = \dot{\boldsymbol{\varepsilon}}^e + \dot{\boldsymbol{\varepsilon}}^p \tag{S8}$$

By adopting the linear hyper-elasticity, the elastic elation is given as follows:

$$\dot{\boldsymbol{\varepsilon}}^e = \utilde{\boldsymbol{E}}^{-1}:\dot{\utilde{\boldsymbol{\sigma}}},\ \ \dot{\utilde{\boldsymbol{\sigma}}} = \utilde{\boldsymbol{E}}:\dot{\boldsymbol{\varepsilon}}^e \tag{S9}$$

where $\utilde{\boldsymbol{E}}$ is the elastic tensor of non-damaged state, and is given by Hooke's law.

First, the isotropic yield surface is considered with:

$$f(\utilde{\boldsymbol{\sigma}}) = \utilde{F}(\utilde{H}) \tag{S10}$$

where $f(\utilde{\boldsymbol{\sigma}})$ is the stress function, and $\utilde{F}(\utilde{H})$ is the isotropic hardening function; i.e. the function of

the isotropic hardening variable $\underset{\sim}{H}$ in the fictitious undamaged configuration. Here, based on the subloading surface concept within the framework of the unconventional elastoplastic theory, it is assumed that the interior of the yield surface in Eq. (S10) is not a purely elastic domain, and plastic deformation may also occur due to the change of the stress inside the yield surface[S11, S12, S13]. Thus, Eq. (5) describes the normal-yield surface.

Next, the subloading-surface is introduced, which always passes through the stress $\underset{\sim}{\boldsymbol{\sigma}}$, and maintains a shape similar to that of the normal-sliding surface. The subloading surface can then be described as:

$$f(\underset{\sim}{\boldsymbol{\sigma}}) = R\underset{\sim}{F}(\underset{\sim}{H}) \tag{S11}$$

where $R(0 \leq R \leq 1)$ is called the normal-yield ratio.

The evolution law of the normal-yield ratio is given by:

$$\dot{R} = U(R)||\dot{\boldsymbol{\varepsilon}}^p|| \quad \text{for} \quad \dot{\boldsymbol{\varepsilon}}^p \neq \mathbf{0} \tag{S12}$$

where $U(R)$ is a monotonically-decreasing function of R and $||\ \ ||$ stands for in this case the magnitude of $\dot{\boldsymbol{\varepsilon}}^p$.

The differentiation of Eq. (S11) with respect to time of the subloading surface while considering Eq.(S12) leads to:

$$\frac{\partial f(\underset{\sim}{\sigma})}{\partial \underset{\sim}{\sigma}} : \dot{\underset{\sim}{\boldsymbol{\sigma}}} = U||\dot{\boldsymbol{\varepsilon}}^p||\underset{\sim}{F} + R\underset{\sim}{F}'\dot{\underset{\sim}{H}} \tag{S13}$$

where

$$\underset{\sim}{F}' \equiv \frac{d\underset{\sim}{F}}{d\underset{\sim}{H}} \tag{S14}$$

By considering the following relation

$$\frac{\partial f(\underset{\sim}{\sigma})}{\partial \underset{\sim}{\sigma}} : \underset{\sim}{\boldsymbol{\sigma}} = R\underset{\sim}{F} \tag{S15}$$

Eq.(S13) can be rewritten as follows:

$$\underset{\sim}{\boldsymbol{n}} : \left[\dot{\underset{\sim}{\boldsymbol{\sigma}}} - \left(\frac{\underset{\sim}{F}'\dot{\underset{\sim}{H}}}{\underset{\sim}{F}} + \frac{U||\dot{\varepsilon}^p||}{R}\right)\underset{\sim}{\boldsymbol{\sigma}}\right] = 0 \tag{S16}$$

Here, $\underset{\sim}{\boldsymbol{n}}$ is the outward normal vector at the stress point on the subloading surface and is given as follows:

$$\underset{\sim}{\boldsymbol{n}} \equiv \frac{\frac{\partial f(\underset{\sim}{\sigma})}{\partial(\underset{\sim}{\sigma})}}{\left\|\frac{\partial f(\underset{\sim}{\sigma})}{\partial(\underset{\sim}{\sigma})}\right\|} \quad (||\underset{\sim}{\boldsymbol{n}}|| = 1) \tag{S17}$$

Now, we adopt the following associated flow rule

$$\dot{\boldsymbol{\varepsilon}}^p = \dot{\lambda}\boldsymbol{n} \tag{S18}$$

where $\dot{\lambda}(\geq 0)$ is a positive plastic multiplier. Substituting Eq. (S18) into Eq. (S16) it is obtained that:

$$\underset{\sim}{\boldsymbol{n}} : \left[\dot{\underset{\sim}{\boldsymbol{\sigma}}} - \left(\frac{\underset{\sim}{F}' f_h \dot{\lambda}}{\underset{\sim}{F}} + \frac{U\dot{\lambda}}{R}\right)\underset{\sim}{\boldsymbol{\sigma}}\right] = 0 \tag{S19}$$

where

$$\underset{\sim}{f_h} \equiv \dot{\underset{\sim}{H}}/\dot{\lambda} \tag{S20}$$

noting the homogeneity of $\dot{\underset{\sim}{H}}$ in degree-one of $\dot{\boldsymbol{\varepsilon}}^p$.

It follows from Eqs (S18) and (S19) that:

$$\dot{\lambda} = \frac{\underset{\sim}{\boldsymbol{n}}:\dot{\underset{\sim}{\boldsymbol{\sigma}}}}{\underset{\sim}{M}^p}, \quad \dot{\underset{\sim}{\boldsymbol{\varepsilon}}}^p = \frac{\underset{\sim}{\boldsymbol{n}}:\dot{\underset{\sim}{\boldsymbol{\sigma}}}}{\underset{\sim}{M}^p}\underset{\sim}{\boldsymbol{n}} \tag{S21}$$

where the plastic modulus $\underset{\sim}{M}^p$ is given by:

$$\underset{\sim}{M}^p \equiv \underset{\sim}{\boldsymbol{n}}:\left[\left(\frac{\underset{\sim}{F}'}{\underset{\sim}{F}}\underset{\sim}{f_h} + \frac{U}{R}\right)\underset{\sim}{\boldsymbol{\sigma}}\right] \tag{S22}$$

The strain rate is described using Eqs (S8), (S9) and (S21) as:

$$\dot{\boldsymbol{\varepsilon}} = \underset{\sim}{\boldsymbol{E}}^{-1}:\dot{\underset{\sim}{\boldsymbol{\sigma}}} + \frac{\underset{\sim}{\boldsymbol{n}}:\dot{\underset{\sim}{\boldsymbol{\sigma}}}}{\underset{\sim}{M}^p}\underset{\sim}{\boldsymbol{n}} = \left(\underset{\sim}{\boldsymbol{E}}^{-1} + \frac{\underset{\sim}{\boldsymbol{n}}\otimes\underset{\sim}{\boldsymbol{n}}}{\underset{\sim}{M}^p}\right):\dot{\underset{\sim}{\boldsymbol{\sigma}}} \tag{S23}$$

The plastic multiplier in terms of strain rate, $\dot{\Lambda}$, is derived with Eq. (S23), as follows:

$$\dot{\Lambda} = \frac{\underset{\sim}{\boldsymbol{n}}:\boldsymbol{E}:\dot{\boldsymbol{\varepsilon}}}{\underset{\sim}{M}^p + \underset{\sim}{\boldsymbol{n}}:\boldsymbol{E}:\underset{\sim}{\boldsymbol{n}}} \tag{S24}$$

Finally, the stress rate is described using Eqs. (S9), (S18) and (S24) as:

$$\dot{\underset{\sim}{\boldsymbol{\sigma}}} = \boldsymbol{C}^{ep}:\dot{\boldsymbol{\varepsilon}} = \left(\underset{\sim}{\boldsymbol{E}} - \frac{\underset{\sim}{\boldsymbol{E}}:\underset{\sim}{\boldsymbol{n}}\otimes\underset{\sim}{\boldsymbol{n}}:\underset{\sim}{\boldsymbol{E}}}{\underset{\sim}{M}^p + \underset{\sim}{\boldsymbol{n}}:\boldsymbol{E}:\underset{\sim}{\boldsymbol{n}}}\right):\dot{\boldsymbol{\varepsilon}} \tag{S25}$$

The loading criterion[S11, S12, S13] is given by:

$$\left.\begin{array}{l}\dot{\boldsymbol{\varepsilon}}^p \neq \boldsymbol{O} \text{ for } \dot{\Lambda} > 0 \\ \dot{\boldsymbol{\varepsilon}}^p = \boldsymbol{O} \text{ for } \dot{\Lambda} \leq 0\end{array}\right\} \tag{S26}$$

The actual functions of the stress function $f(\underset{\sim}{\boldsymbol{\sigma}})$, the isotropic hardening function $\underset{\sim}{F}(\underset{\sim}{H})$, and other evolution rules are given in the sequel.

In this study, the von Mises yield condition is adopted for Ti_2AlC according to:

$$f(\underset{\sim}{\boldsymbol{\sigma}}) = \sqrt{\frac{3}{2}}||\underset{\sim}{\boldsymbol{\sigma}}'|| \tag{S27}$$

where $\underset{\sim}{\boldsymbol{\sigma}}'$ is the deviatoric stress tensor.

Meanwhile, the isotropic hardening function[S12, S13] is described by:

$$\underset{\sim}{F}(\underset{\sim}{H}) = \underset{\sim}{F}_0[1 + h_1\{1 - \exp(-h_2\underset{\sim}{H})\}], \ \underset{\sim}{F}' = \underset{\sim}{F}_0 h_1 h_2\, exp(-h_2\underset{\sim}{H}) \tag{S28}$$

hence,

$$\dot{\underset{\sim}{H}} = \sqrt{\frac{2}{3}}||\dot{\boldsymbol{\varepsilon}}^p|| = \dot{\lambda}\underset{\sim}{f_h}, \ \underset{\sim}{f_h} = \sqrt{\frac{2}{3}} \tag{S29}$$

where $\underset{\sim}{F}_0$, h_1 and h_2 are the material constants. The hardening function F in Eq. (S28) increases from the initial value $\underset{\sim}{F}_0$ by the equivalent plastic strain and saturates when it reaches the maximum value

$(1+h_1)\underset{\sim}{F}_0$. $\underset{\sim}{F}_0$ corresponds to the initial yield stress in conventional elastoplastic models.

The following function is applied to the evolution rule of normal-sliding ratio R:

$$U(R) = u\cot\left(\frac{\pi}{2}\frac{\langle R-R_e\rangle}{1-R_e}\right) \tag{S30}$$

where < > denotes Macaulay's bracket. R_e and u are material parameters describing the elastic limit and smoothness of stress-strain curve, respectively.

Now, the damage evolution concept used is explained. In the newly developed constitutive model, it is assumed that the relationship between the cohesive force (traction) σ and the crack opening displacement w by tensile fracture can be expressed by an exponential function according to:

$$\sigma = \sigma_F \exp\left(-\frac{\sigma_F}{G_C}w\right) \tag{S31}$$

where σ_F and G_C are the one-dimensional tensile fracture strength and the critical energy release rate.

The crack opening displacement is described by the relationship between the displacement and the related strain ε as follows:

$$w = (\varepsilon - \varepsilon_0)h \tag{S32}$$

where ε_0 is the strain at damage initiation, h denotes the length of the finite element (characteristic length)[S14] Further, the cohesive force σ applied to the fracture surface is equivalent to the stress. Thus, by representing ε in the damage variable $D(\varepsilon)$ as the maximum strain $\bar{\varepsilon} \geq 0$ observed in the deformation history, the damage variable $D(\bar{\varepsilon})$ can be expressed as follows:

$$D(\bar{\varepsilon}) = 1 - \frac{\varepsilon_0}{\bar{\varepsilon}}\exp\left(-\frac{\sigma_F h}{G_C}(\bar{\varepsilon}-\varepsilon_0)\right) \tag{S33}$$

Eqs (S31) till (S33) have been applied to damage phenomena of ceramics and its validity has been verified[S15-S17].

In general, the equivalent strain (which is a scalar) is used in the isotropic damage model applied to multi-dimensional problems. In the same manner as in previous studies [S15-S17], the equivalent strain ε_{eq} based on the modified von Mises equation is adopted and reads:

$$\varepsilon_{eq} = \frac{k-1}{2k(1-2\nu)}I_1 + \frac{1}{2k}\sqrt{\left(\frac{k-1}{1-2\nu}I_1\right)^2 + \frac{12k}{(1+\nu)^2}J_2} \tag{S34}$$

where k is the ratio between the tensile and compressive strength I_1 is the first invariant of the strain tensor, while J_2 is the second invariant of the deviatoric strain tensor.

To describe the damage history dependency of damage variable, the equivalent strain ε_{eq} is used denoted as κ. By applying the maximum value of the equivalent strain κ to the deformation history, the damage variable $D(\kappa)$ can be expressed with:

$$D(\kappa) = 1 - \frac{\kappa_0}{\kappa}\exp\left(-\frac{\sigma_F h}{G_C}(\kappa-\kappa_0)\right) \tag{S35}$$

where κ_0 is the equivalent strain at damage initiation.

The loading criterion and the judgement of damage are described by:

$$\left.\begin{array}{ll}\dot{\kappa} = <\dot{\varepsilon}_{eq}> & \text{if } \kappa = \varepsilon_{eq} \\ \dot{\kappa} = 0 & \text{if } \kappa > \varepsilon_{eq}\end{array}\right\} \tag{S36}$$

The evolution of damage variable is given by Eq. (S35) as:

$$\left.\begin{array}{ll}\dot{D} = \frac{\partial D(\kappa)}{\partial \kappa}\frac{\partial \kappa}{\partial \varepsilon_{eq}}\frac{\partial \varepsilon_{eq}}{\partial t} = \left(\frac{1}{\kappa} + \frac{\sigma_F h}{G_C}\right)(1-D)\dot{\varepsilon}_{eq} & \text{for } <\dot{\varepsilon}_{eq}> \neq 0 \\ \dot{D} = 0 & \text{for } <\dot{\varepsilon}_{eq}> = 0\end{array}\right\} \tag{S37}$$

By substituting Eqs (S25) and (S37) into Eq. (S7), the current stress rate is calculated considering elastoplastic deformation and damage. It should be noted that the subloading surface model [S11, S12, S13] describes the elastic-plastic transition fulfilling always the smoothness condition. Then, it possesses the automatic controlling function to attract the stress to the yield surface in the plastic deformation process so that the stress is automatically pulled-back to the yield surface when it goes out from the yield surface by finite loading increments in numerical calculation. The 3-dimensional finite element modelling of wedge splitting test and typical mechanical response of the EPD constitutive model are shown in Fig. 6S. Further, parameters of EPD constitutive model used for the inverse analysis is listed in Table S1.

Supplementary references

S1. Peterlik, H., Roschger, P., Klaushofer, K. & Fratzl, P., From brittle to ductile fracture of bone, *Nature Mater.* 5, 52 – 55 (2006).

S2. Thompson, J.B, Kindt, J.H., Drake, B., Hansma, H.G., Morse, D.E. & Hansma, P.K., Bone indentation recovery time correlates with bone reforming time, *Nature* 414, 773 – 776 (2001).

S3. Zioupos, P. & Currey, J.D., The extent of microcracking and the morphology of microcracks in damaged bone, *J. Mater. Sci.* 29, 978 – 986 (1994).

S4. Nalla, R.K., Kinney, J.H. & Ritchie, R.O., Mechanistic fracture criteria for the failure of human cortical bone, *Nature Mater.* 2, 164 – 168 (2003).

S5. Evans, A.G. and Graham, L.J., A model for crack propagation in polycrystalline ceramics, *Acta Metallugica*, 23, 1303 – 1312 (1975).

S6. M.G.R.Sause et al., Quantification of metallic coating failure on carbon fiber reinforced plastics using acoustic emission, Surface & Coatings Technology, 204, 300 – 308 (2009).

S7. Irwin,G.R., Analysis of stresses and strains near the end of a crack traversing a plate, *J. App. Mech.*, 24, 361 – 364 (1957).

S8. Barsoum M.W., The $M_{(N+1)}AX_{(N)}$ phases: a new class of solids; thermodynamically stable nanolaminates, *Prog. Solid. State. Chem.* 28, 201-281 (2000).

S9. Barsoum M.W. & Radovic, M., Elastic and mechanical properties of the MAX phases, Ann. Rev.

Mater. Res., 41, 195-227 (2011).

S10. Lemaitre, J.A., A course on damage mechanics. *Springer, Heidelberg*, (1992)

S11. Hashiguchi, K., Foundations of elastoplasticity: Subloading surface model, *Springer*, Third edition (2017).

S12. Hashiguchi, K., Constitutive equations of elastoplastic materials with elastic-plastic transition. *J. Appl. Mech.* (ASME), 47, 266-272, (1980)

S13. Hashiguchi, K., Subloading surface model in unconventional plasticity, *Int. J. Solds Struct*, 25, 917-945, (1989)

S14. Oliver, J., A consistent characteristic length or smeared cracking models. *Int. J. Numer. Methods Eng.*, 28, 461-474 (1989)

S15. Ozaki S, Osada T, Nakao W. Finite element analysis of the damage and healing behaviour in self-healing ceramic materials. *Int. J. Solids Struct.*, 100, 307-318, (2016)

S16. Ozaki S, Aoki Y, Osada T, Takeo K, Nakao W. Finite element analysis of fracture statistics of ceramics: Effects of grain size and pore size distributions. *J. Am. Ceram. Soc.*, 101,3191-3204, (2018)

S17. Takeo, K., Aoki, Y., Osada, T., Nakao, W., Ozaki, S., Finite element analysis of the size effect on ceramic strength, *Materials*, 12, 2885 (2019)

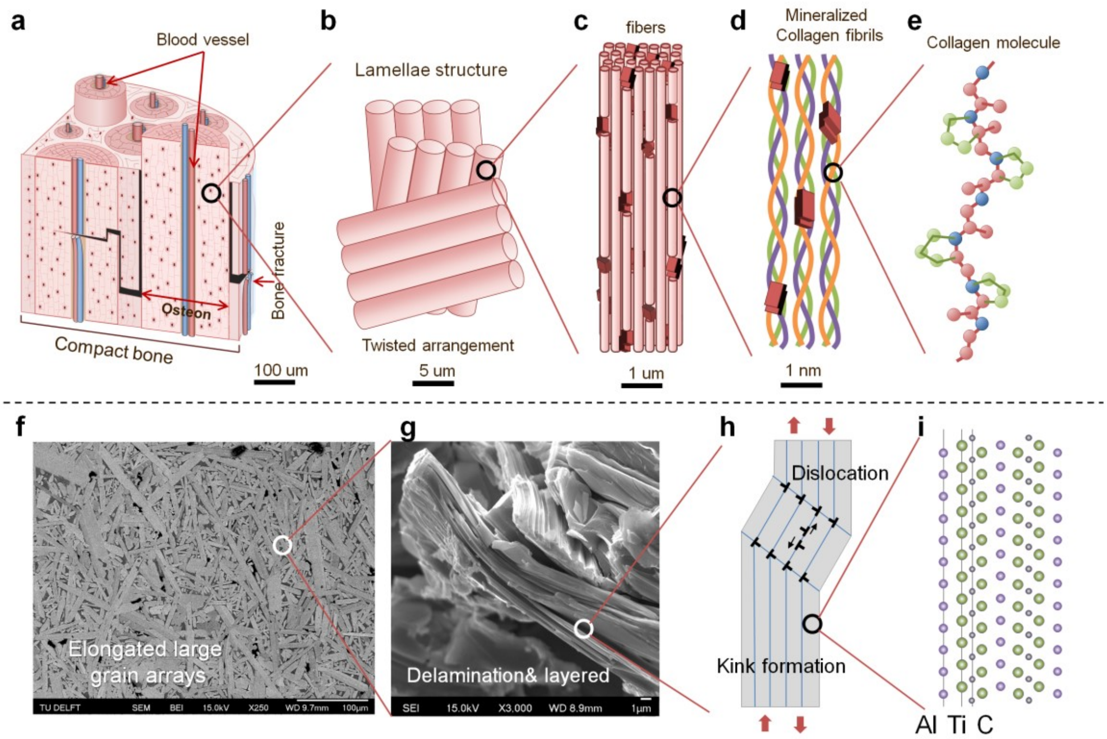

Figure S1 Hierarchical microstructures in Ti_2AlC like in human or animal bone. a Compact bone structure including osteon. **b** Twisted arrangement of fibers. **c** Fibers composed of: **d** Mineralized collagen fibrils, and **e** collagen molecules. **f** Microstructure composed of large elongated grainsTi_2AlC randomly-distributed. **g** Delamination of layered structure within the grain. **h** Illustration of kink band formation due to dislocation motion. **i** Atomic level of layered crystal structure of Al, Ti, and C in Ti_2AlC.

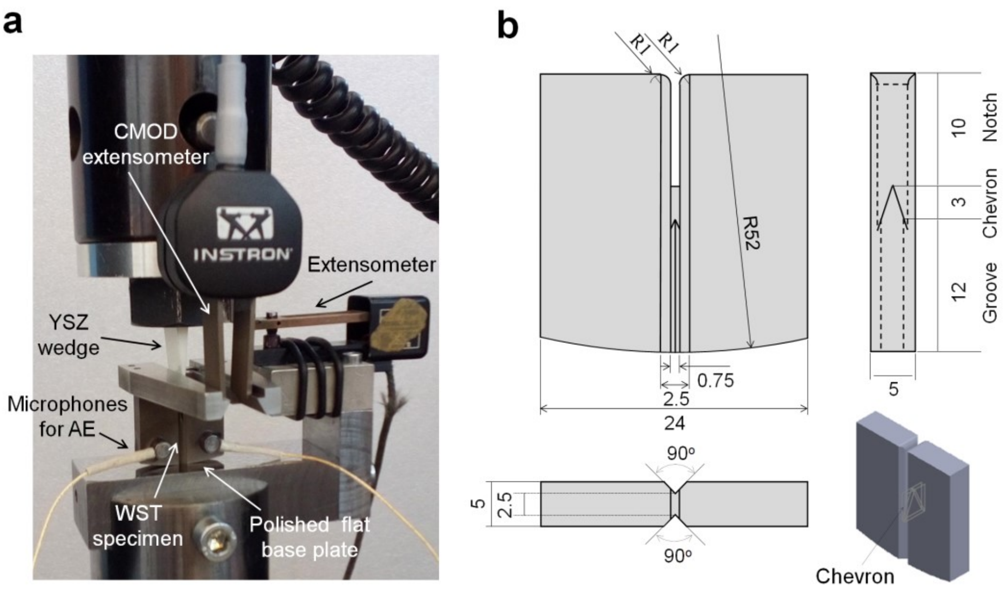

Figure S2 Wedge Splitting Test (WST) setup for controlled crack growth: **a** Full set up of wedge splitting test including specimen, wedge, legs clip gauge and acoustic emission microphones. **b** Geometry of the WST specimen including straight notch, chevron notch, and 90º groove for guiding crack propagation.

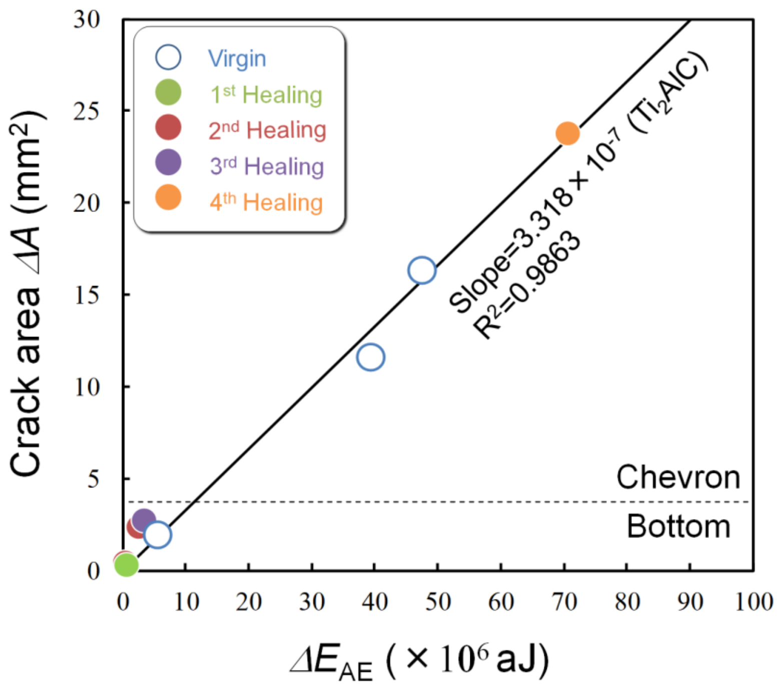

Figure S3 Relationship between cumulative acoustic emission energy and crack area.

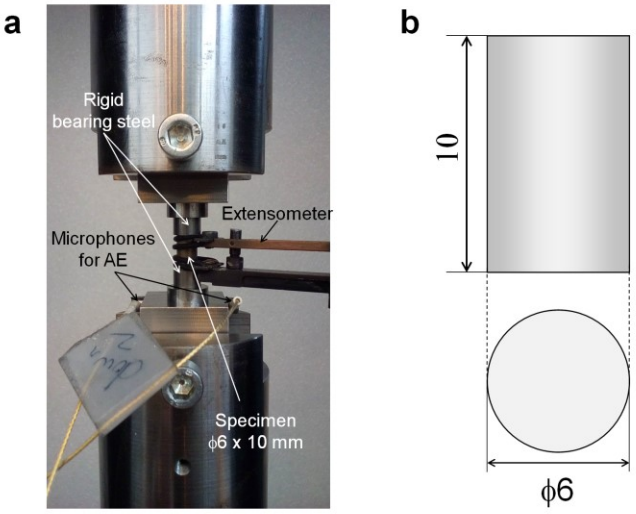

Figure S4 Cyclic compression test: a Full set up of compression test including specimen, extensometer and acoustic emission microphone. **b** Geometry of cylindrical specimen.

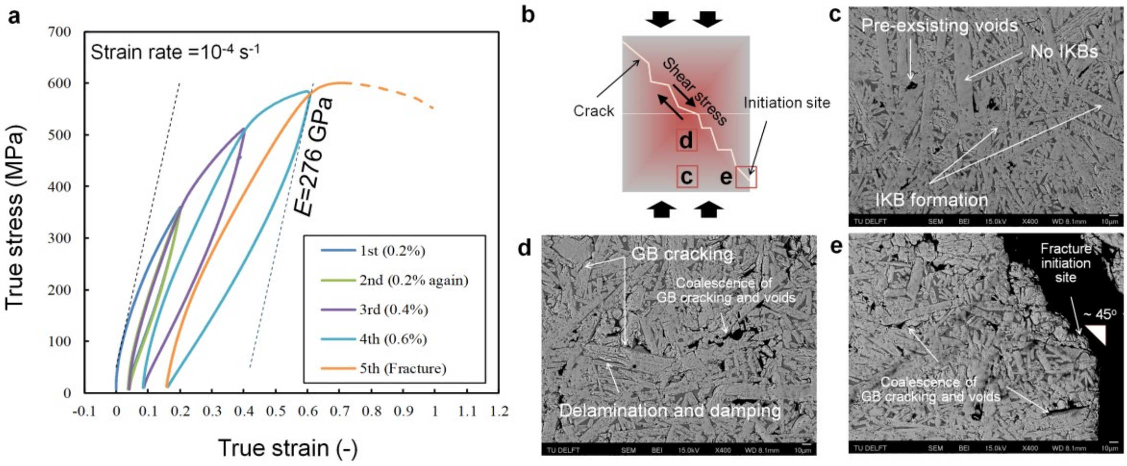

Figure S5 Cyclic compressive deformation: a True stress-strain curves. **b** Illustration of the shear strain distribution and cracking in the specimen. **c, d and e** Typical deformation mechanism in MAX-phase ceramic, **c** Incipient Kink Band(IKB) formation, **d** delamination and grain boundary (GB) cracking, and **e** fracture initiation site with a 45° inclination at the region c, d and e indicated in **b**, respectively.

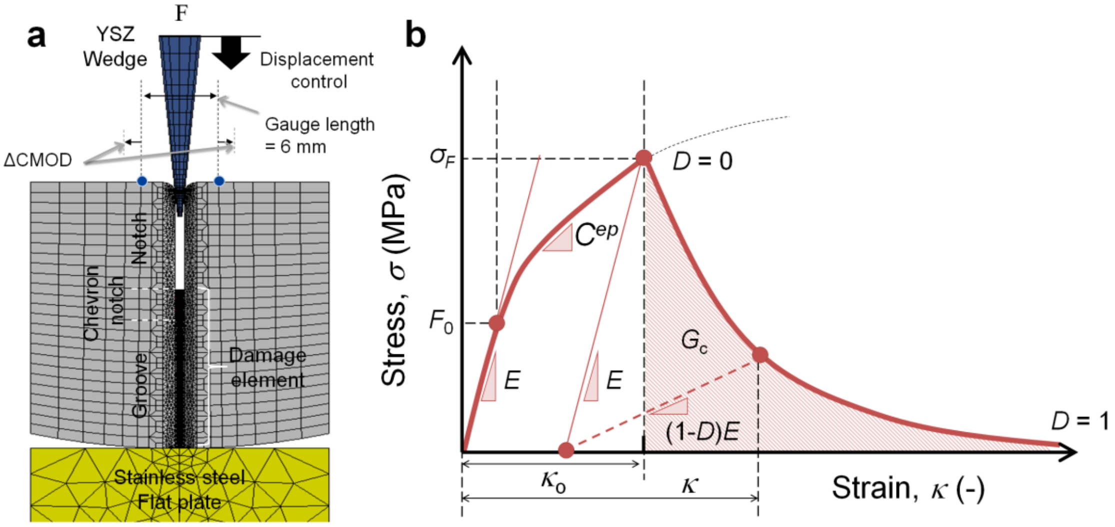

Figure S6 3D Finite Element Model: a Model for the wedge-splitting test setup. **b** Cohesive-force embedded damage model describing elastic-plastic-damage behavior in MAX phase metallo-ceramics.

Table S1 Parameters of elastoplastic-damage constitutive model:

Symbol	Value	Determination
E	276 [GPa]	From compressive test
v	0.19 [-]	From compressive test
F_0	125 [MPa]	From compressive test
h_1	3.9 [-]	From compressive test
h_2	730 [-]	From compressive test
R_e	0.8 [-]	From compressive test
u	1000 [1/s]	From compressive test
σ_F	- [MPa]	From inverse FE analysis
G_c	- [J]	From inverse FE analysis
h_e	0.05 [mm]	From length of element
k_0	- [-]	From inverse FE analysis
